# A Quantitative Synthesis of Mercury in Commercial Seafood and Implications for Exposure in the United States

**DOI:** 10.1289/ehp.1205122

**Published:** 2012-06-25

**Authors:** Roxanne Karimi, Timothy P. Fitzgerald, Nicholas S. Fisher

**Affiliations:** 1 School of Marine and Atmospheric Sciences, Stony Brook University, Stony Brook, New York, USA; 2 Environmental Defense Fund Oceans Program, Washington, DC, USA

**Keywords:** aquaculture, consumption advisory, contaminants, fisheries, Seafood Hg Database, seafood safety

## Abstract

Background: Mercury (Hg) is a toxic metal that presents public health risks through fish consumption. A major source of uncertainty in evaluating harmful exposure is inadequate knowledge of Hg concentrations in commercially important seafood.

Objectives: We examined patterns, variability, and knowledge gaps of Hg in common commercial seafood items in the United States and compared seafood Hg concentrations from our database to those used for exposure estimates and consumption advice.

Methods: We developed a database of Hg concentrations in fish and shellfish common to the U.S. market by aggregating available data from government monitoring programs and the scientific literature. We calculated a grand mean for individual seafood items, based on reported means from individual studies, weighted by sample size. We also compared database results to those of federal programs and human health criteria [U.S. Food and Drug Administration Hg Monitoring Program (FDA-MP), U.S. Environmental Protection Agency (EPA)].

Results: Mean Hg concentrations for each seafood item were highly variable among studies, spanning 0.3–2.4 orders of magnitude. Farmed fish generally had lower grand mean Hg concentrations than their wild counterparts, with wild seafood having 2- to12-fold higher concentrations, depending on the seafood item. However, farmed fish are relatively understudied, as are specific seafood items and seafood imports from Asia and South America. Finally, we found large discrepancies between mean Hg concentrations estimated from our database and FDA-MP estimates for most seafood items examined.

Conclusions: The high variability in Hg in common seafood items has considerable ramifications for public health and the formulation of consumption guidelines. Exposure and risk analyses derived from smaller data sets do not reflect our collective, available information on seafood Hg concentrations.

Human mercury (Hg) exposure from seafood consumption and its attendant risks are difficult to estimate and are often the subject of intense debate. However, there is broad recognition of the need for large-scale information on Hg concentrations in marine fish and shellfish in order to better understand and control Hg exposure and risk (National Research Council 2000). Although U.S. seafood consumption has plateaued in recent years, global seafood demand is on the rise [Food and Agriculture Organization of the United Nations (FAO) 2010; National Marine Fisheries Service (NMFS) 2011a]. Fish and shellfish are important sources of lean protein and other nutrients, including essential omega-3 fatty acids, which confer important health benefits (Albert et al. 2002; Huynh and Kitts 2009; Simopoulos 1991; Siscovick et al. 1995). However, all seafood also contains Hg, primarily in the form of methylmercury (MeHg). In sufficient doses, MeHg can cause adverse neurodevelopmental (Myers et al. 2009; Oken et al. 2005; Trasande et al. 2005), cardiovascular (Grandjean et al. 2004), and immunological health effects (Gardner et al. 2010). Because most human exposure to MeHg is through seafood consumption [International Programme on Chemical Safety (IPCS) 1990, 1991; National Research Council 2000; United Nations Environment Programme 2002], it is critical to have reliable estimates of Hg concentrations in seafood items in order to confidently identify those that are low in Hg. Such efforts will better inform estimates of exposure and risk and help consumers make decisions about the types and quantities of seafood that are both safe to eat and nutritionally beneficial.

Seafood Hg concentrations can be highly variable, even within the same species (National Research Council 2000; Sunderland 2007). Although hundreds of individual studies collectively have monitored fish Hg concentrations around the world, we still have an incomplete understanding of general Hg patterns, particularly in commercial fish and shellfish from marine waters (Chen et al. 2008). Moreover, our knowledge of the extent of Hg variability is limited. Aggregating data from individual studies is necessary to obtain a clearer understanding of general patterns in Hg content of commercial fish. To date, the largest, most well-known existing databases on Hg content in U.S. commercial fish were developed by federal government agencies [NMFS, Food and Drug Administration (FDA)]. However, the NMFS study from the 1970s (Hall et al. 1978) is relatively outdated, and the FDA Monitoring Program (FDA 2011; FDA-MP 2011) contains smaller sample sizes and fewer species. In contrast, data from intensive, small-scale studies that focus on obtaining large sample sizes of a specific taxon are less susceptible to random sampling error and are likely to yield better estimates of central tendency. Such smaller, intensive studies are common within the scientific literature (e.g., Adams and McMichael 2007; Burger and Gochfeld 2006) but typically are not integrated into larger analyses of exposure and risk. Finally, federal databases, particularly the NMFS study, may not accurately reflect Hg concentrations of imported fish, even though the amount of imported, edible seafood consumed in the United States is increasing (NMFS 2010). Imports now account for > 80% of the seafood eaten in the United States (NMFS 2011a). Thus, the inclusion of Hg data for imported seafood would fill a crucial knowledge gap. Combining data from government and academic sources would allow for more precise estimates of Hg concentrations in U.S. imported and domestic seafood items using the broadest available knowledge base.

We examined patterns of Hg concentrations in U.S. commercial seafood items using, to our knowledge, the largest compilation of available academic and agency data to date. Our overarching goal was to examine long-standing questions about the patterns of seafood Hg concentrations and their variability. Our Seafood Hg Database (Karimi 2012) aggregates Hg measurements of hundreds of seafood items from federal and state agencies, as well as from smaller, more intensive studies in the scientific literature. Our specific goals were to reliably identify low-Hg and high-Hg fish, and to identify the seafood items and geographic regions for which further study is most needed. We also compared Hg concentrations in farmed seafood items relative to concentrations in their wild seafood counterparts. Finally, we compared Hg concentrations for individual seafood items to those summarized from the FDA-MP (2011). FDA-MP data are commonly used for risk assessment and exposure estimates (Ginsberg and Toal 2009; Sunderland 2007; Tran et al. 2004) and in the development of state-level consumption advice for consumers (e.g., State of Maryland 2011; State of Minnesota 2011). At least one previous study has compared FDA-MP data to those from independent studies in order to better estimate Hg intake in the United States (Sunderland 2007). Our study builds on this approach by synthesizing a much larger aggregation of available data to better characterize Hg variability and assess the current state of knowledge of seafood Hg content. Ideally, these improved estimates of Hg concentrations in commercial fish will help enable more accurate assessments of potential exposure and inform both public health programs and the public itself regarding the types and amounts of fish that are safe to eat.

## Methods

*Data gathering and inclusion criteria.* Our guiding principle for building the Seafood Hg Database (Karimi 2012) was to focus on fish and shellfish from sources that could reasonably be sold in the United States. Our database was developed to reflect the range of possible Hg levels for seafood items considered the top contributors to human Hg exposure in the United States because they are relatively high in Hg and/or they constitute relatively large shares of the U.S. seafood market (top 51 Hg contributors defined by Groth 2010). Detailed taxonomic and geographic harvest information is often lacking or incorrect in the seafood marketplace (Jacquet and Pauly 2008). Thus, our database does not model the exact composition of the U.S. seafood market. Rather it reflects the range of seafood species and seafood Hg concentrations that are available to U.S. seafood consumers.

Data were gathered from federal and state government reports and from peer-reviewed scientific literature. We obtained data from federal and state government agencies that either made their fish tissue monitoring results publically available online [e.g., State of North Carolina (2011); State of Virginia, Department of Environmental Quality (2009); U.S. Environmental Protection Agency (EPA) National Coastal Assessment (U.S. EPA 2008); U.S. FDA raw data (FDA 2011)] or provided data upon request (e.g., State of Delaware, State of Hawaii). In addition, we searched for published, peer-reviewed papers indexed in the Web of Science (Thomson Reuters, New York, NY) before 15 December 2010. We conducted literature searches for individual seafood items based on seafood varieties listed as the top 51 Hg contributors to the U.S. population (Groth 2010). Search terms included “mercury” and the common names of these fish or shellfish (e.g., “mercury AND salmon”) [see Supplemental Material, Search Terms for Table S2 (http://dx.doi.org/10.1289/ehp.1205122)].

From the data gathering and search results, we included select studies or select data from studies on fish and shellfish from sources that were likely to enter the U.S. seafood market. We included data on edible portions (fillet or whole fish) of any fish or shellfish species likely to be included in the top 51 seafood varieties (e.g., “redfish” were included with “ocean perch”) based on federal commercial fisheries landings (fisheries landed and sold in the United States) and seafood import statistics (NMFS 2007b). Hg concentrations in whole fish can be lower than concentrations in fillets (Goldstein et al. 1996), probably because Hg is primarily associated with muscle tissue. Thus, the inclusion of data based on fillets as well as whole fish, which are common in the market particularly for smaller fish such as anchovies, may underestimate fish Hg content relative to those based on fillets only. We classified seafood items as being from domestic or imported sources based on geographic locations specified in the original study. We assumed that all marine fish caught commercially from domestic waters were relevant to the U.S. market. Data for a given fish or shellfish species collected from market basket studies or direct harvest from countries outside of the United States were included only if at least 5% of all imports of that species into the U.S. fisheries market (by volume) were from that country according to NMFS import statistics as of 2010 (NMFS 2011b). In addition, imported seafood items that did not meet this criterion were included if the samples were collected from water bodies connected to other countries that meet this criterion. Highly migratory fish caught from major ocean basins (tuna, shark, and swordfish) were included regardless of country of origin.

Of the top 51 seafood varieties, < 10 are freshwater fish. For most freshwater items collected from domestic waters, we included data from the Great Lakes because the Great Lakes are the main sources of these species to the market (NMFS 2011c). We did not include salmon species from the Great Lakes because the commercial catch of salmon from the Great Lakes has been negligible for at least one decade (Baldwin et al. 2009). For striped bass (*Morone saxatilis*), we included data for wild fish only from Atlantic states because commercial fisheries do not exist for this species in the Gulf of Mexico or Pacific coast (NMFS 2007a). For catfish, carp, and perch, we included fish collected from Atlantic or Gulf Coast states that report commercial landings of these fish (NMFS 2007a), excluding samples from interior or landlocked freshwater sources. Data for farmed species of commercial freshwater fish were included if the fish were specifically raised for consumption (e.g., farmed catfish), and the fish were of market size (vs. juvenile fish from hatcheries) and were fed conventional feed (e.g., Berntssen et al. 2010).

*Exclusion criteria.* We screened approximately 1,000 government monitoring programs and peer-reviewed academic studies for inclusion. Upon critically examining each study, we excluded entire data sets—or select data from studies—based on one or more of the following criteria:

Data resulting from experimental Hg exposures.
Data on fish or shellfish that are not a primary source of commercial fish to U.S. consumers, based on the geographic location of collection.
Studies that were not written in the English language.
Data that were repeated from another source already included the database; for example, data repeated in review papers as well as original papers, or data repeated in aggregate federal government databases (e.g., U.S. EPA National Listing of Fish Advisories) and original state data sources (e.g., State of North Carolina). Duplicate entries were routinely screened for and excluded from all calculations.
Data for fish from locations with known point source Hg contamination or associated fisheries closures.
Data for young-of-year fish (born within the past year). However, we included Hg values from other smaller body size fish that may be excluded from the U.S. market because of catch restrictions. Hg concentrations tend to be lower in small fish than in larger fish of the same species, thus may lead to underestimates of the true average of Hg values in U.S. commercial fish.
Studies conducted by nongovernmental organizations, public interest groups, or news media that were not peer-reviewed or incorporated into government monitoring efforts.
Studies that did not report the necessary Hg data (raw data, or arithmetic mean Hg or MeHg concentration and sample size). For example, we excluded studies that presented Hg concentrations in a graph or as a range, geometric mean, or median. Geometric means and medians were rarely reported in the literature. Therefore, we included only arithmetic mean Hg concentrations, or we calculated arithmetic means based on raw data when reported.
Data from areas with no commercial fishing activity, such as no-take marine reserves and national parks (e.g., Rencz et al. 2003; Wyn et al. 2009).

*Data extraction.* We extracted mean Hg concentrations (parts per million, wet weight), sample size, and geographic location for each seafood item reported in each study. Approximately 40% of the included sources reported SDs or SEs. Thus, analyses requiring SDs or SEs would exclude the bulk of the data set. Therefore, we focused on examining mean Hg concentrations in the interest of including the range of Hg concentrations for each seafood item using the largest possible data set. We extracted total Hg values whenever possible, but we used MeHg values when they were reported instead of total Hg. Approximately 95% of total Hg in fish muscle tissue occurs in the form of MeHg (Bloom 1992). Therefore, we assumed that MeHg concentrations are similar to total Hg concentrations. Nevertheless, because MeHg concentrations are lower than total Hg, our calculated, grand mean Hg concentrations for certain seafood items may be slightly lower than if they were based solely on total Hg concentrations. Hg values reported as dry weight concentrations were converted to wet weight concentrations according to moisture content, if reported, or by assuming 80% water content. When Hg concentrations were reported as nondetects (approximately < 10% of all database entries), we entered values as one-half the detection limit from the study (Clarke 1998); when detection limits were not reported, these values were excluded.

When a study reported multiple mean Hg values for a given seafood item (e.g., Hall et al. 1978), we calculated a weighted mean using sample size for the mean as the weight. When a study reported multiple Hg values for a given seafood item but did not provide sample sizes for individual values (e.g., Cossa et al. 1992; Deshpande et al. 2009; Jackson 1991), we assumed sample sizes were equivalent across values. Thus, overall means calculated from these studies were not weighted.

*Data analysis.* We calculated an aggregate, grand weighted mean (*H^-^g^-^_w_*) for each seafood item based on means weighted by sample size across studies


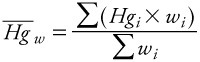
[1]

where *Hg_i_* is the *i*th reported mean and *w_i_* is the weight (reported sample size) of the *i*th observation. We estimated variability of Hg in seafood items by calculating a weighted grand SD, corresponding to the grand mean. The Seafood Hg Database comprises mean Hg values reported by individual studies as observations, as opposed to raw Hg data values. By definition, the SD of sample means is the SE of the global distribution of Hg values. Therefore we estimated the weighted SE (SE_w_) of the distribution underlying the grand mean using the formula for the weighted SD,


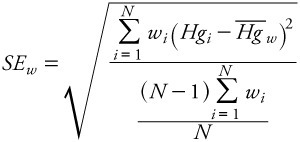
[2]

where *N* is the number of studies from which mean Hg values were collected. To obtain the weighted SD (SD_w_) of the global distribution, we multiplied SE_w_ by the square root of average sample size across studies for each seafood item, yielding the formula


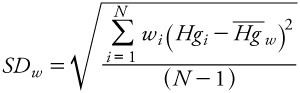
[3]

Monte Carlo simulations tested for potential bias of Equation 3 using hypothetical data approximating the composition of the database. Specifically, we simulated a true standard deviation of the global distribution using random numbers drawn from normal and lognormal distributions, where *w_i_* ranged from 2 to 100 and *N* ranged from 50 to 300. Tests of 10,000 replicates demonstrated that Equation 3 was an unbiased estimator of the true standard deviation of the global distribution and was insensitive to both the type of distribution used and variation in sample size (data not shown).

We calculated the *a*) grand mean, *b*) grand SD, *c*) range (minimum and maximum reported means), *d*) coefficient of variation (CV), and *e*) total number of samples across all studies for each seafood item name searched (e.g., salmon), as well as for seafood items with higher taxonomic resolution within the search results (e.g., Atlantic salmon) and for broader taxonomic categories for specific analyses. Thus, results are presented for a larger number of seafood groups than the original top 51 seafood items from the search. We compared our findings with summarized Hg data accessed from the FDA-MP (2011) on 15 September 2011 for seafood items for which direct comparisons were possible given available data (58 seafood items). In some cases, seafood items were grouped together into larger seafood categories, which often included multiple taxa. For example, for direct comparison with Hg concentrations for “crab” reported by the FDA-MP, we grouped together blue crab, king crab, and snow crab data. Formal parametric statistical comparisons, such as analysis of variance, were not possible for our analyses because the database is composed of aggregate mean Hg values instead of raw data. Thus, unknown distributions of the underlying Hg data, together with unequal sample sizes for the comparisons of interest, made statistical comparisons inappropriate for our study. Finally, we calculated the percentage of studies reporting a mean Hg concentration exceeding the FDA action level (1 ppm) and the U.S. EPA human health criterion (0.3 ppm) for seafood items with relatively higher taxonomic resolution when possible in order to yield more detailed results than those from broader seafood categories. The FDA action level for MeHg of 1.0 ppm represents the threshold above which the agency can take legal action (e.g., removing the product from the marketplace) (FDA 2007). The U.S. EPA MeHg criterion of 0.3 ppm represents the fish tissue concentration that should not be exceeded for safe consumption of sport-caught fish in local waters based on average consumption (U.S. EPA 2001).

To compare farmed items to wild-caught items within the same seafood category, we focused on species with established or emerging, rather than nascent, farming or ranching industries. For some seafood categories, the species composition of farmed and wild items was not identical. For example, wild-caught catfish include channel catfish, blue catfish, and brown bullhead, whereas farmed catfish include channel catfish and striped catfish. We designated individual data as farmed or wild according to information from original studies. When farmed or wild status was not reported, as with some market basket studies, we made assumptions based on FAO fisheries statistics for individual species (FAO 2011). Specifically, we assumed that lake trout were wild-caught and rainbow trout were farmed. For eel species from market studies, we assumed that Japanese eel (*Anguilla japonica*) were farmed and European conger eel were wild caught. Finally, we assumed that Atlantic salmon from market studies in North America and Europe were farmed unless otherwise specified, given the endangered status of wild Atlantic salmon.

## Results

*Overview of the Seafood Hg database.* The Seafood Hg Database contains approximately 300 unique data sources [see Supplemental Material, Table S1 (Summary of Hg concentrations across studies in commonly consumed seafood items in the U.S.) and Table S2 (Seafood Hg Database) (http://dx.doi.org/10.1289/ehp.1205122); the Seafood Hg Database and any further updates are also available from Karimi (2012)]. In contrast with other well-known compilations of U.S. seafood Hg data [the FDA-MP (2011), the NMFS report (Hall et al. 1978), and combined U.S. EPA fish monitoring programs from different regions (e.g., Environmental Monitoring and Assessment Program, Regional Environmental Monitoring and Assessment Program, National Coastal Assessment)], the Seafood Hg Database includes data from both academic and government data sources (approximately 50% of observations from each source type). In addition, the Seafood Hg Database contains large amounts of data on imported fish and shellfish (43% of observations, 21% excluding market studies outside of the United States for which exact seafood origin is uncertain).

*Variability, patterns, and information gaps.* We observed relatively high variability in Hg concentrations for individual seafood items. Mean Hg concentrations reported across studies for a given seafood item spanned 0.3 to 2.4 orders of magnitude (for tilefish from the Gulf of Mexico and tuna, fresh/frozen, respectively), with a mean of 1.3 orders of magnitude [see Supplemental Material, Table S1 (http://dx.doi.org/10.1289/ehp.1205122)]. CVs for individual seafood items ranged from 0.22 (tilefish from the Gulf of Mexico) to 15.42 (softshell clams), with a mean CV of 3.0. We found high variability in Hg content for both broadly defined seafood categories composed of multiple species (e.g., shark, tuna, shrimp), as well as for individual species (e.g., blue crab, *Callinectes sapidus*).

**Figure 1:**
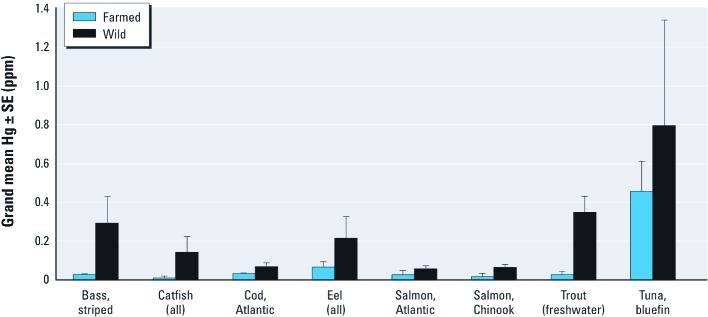
Grand mean Hg ± SE in farmed and wild seafood items by category.

Hg concentrations of wild seafood items were higher than those of farmed items in the same seafood category for all eight seafood categories included in this comparison (Figure 1). Grand mean Hg concentrations for wild items were 2–12 times higher than mean concentrations for farmed counterparts. For example, mean Hg for wild catfish was 12 times higher than mean Hg for farmed catfish. Both wild and farmed seafood items can have low minimum mean Hg concentrations [e.g., 0.005 and 0.008 for wild and farmed catfish, respectively; see Supplemental Material, Table S1 (http://dx.doi.org/10.1289/ehp.1205122)]. However, wild seafood items generally had higher maximum mean Hg concentrations than farmed seafood items within the same seafood category (e.g., 0.714 and 0.030 for wild and farmed catfish, respectively). Finally, we found that except for Atlantic salmon, farmed seafood items are relatively understudied compared with their wild counterparts, based on the total number of samples for each group (see Supplemental Material, Table S1).

**Figure 2:**
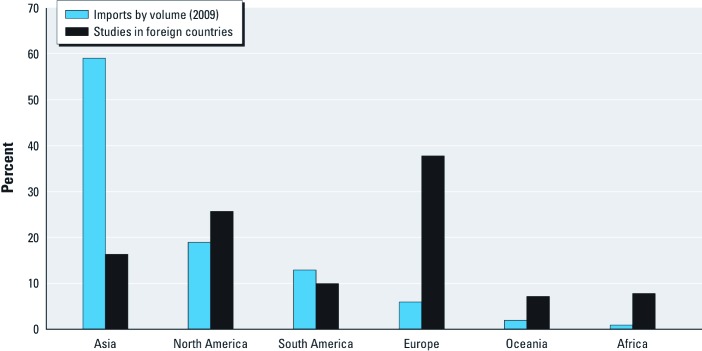
Comparison of the percentage of all seafood imports into the United States by volume in 2009 according to region (NMFS 2010) with the percentage of all non-U.S. studies in the Seafood Hg Database by region conducted in foreign countries. Studies from foreign countries exclude market basket studies, and percent imports do not account for re-exportation of imported fish.

Our analysis indicated that seafood Hg is understudied in some of the world’s most important fisheries. We compared the percentage of studies in the database conducted in major regions in the world (excluding market basket studies) to the percentage of U.S. imports from those regions (NMFS 2010). Hg in seafood from Asia and South America were understudied, whereas Hg in seafood from North America (excluding the United States) and Europe were well studied, relative to the percent imports from those regions (Figure 2). For example, approximately 60% of seafood imported into the United States is from Asia, but only 16% of non-U.S. studies were conducted in Asia. The most studied seafood items, based on the total number of samples measured across studies, include both high-Hg items (0.6 to ≥ 1 ppm) such as shark (grand mean Hg, 0.882 ppm; 3,722 samples) as well as moderate-Hg items (0.3–0.59 ppm) such as tuna (0.450 ppm; 3,780 samples) and low-Hg items (0–0.29 ppm) such as oysters (0.020 ppm; 5,310 samples) [see Supplemental Material, Table S1 (http://dx.doi.org/10.1289/ehp.1205122)]. The least studied items included monkfish (0.174 ppm; 92 samples) and haddock (0.164 ppm; 226 samples) among items with low to moderate Hg, and tilefish (all, 0.883 ppm; 109 samples) and orange roughy (0.513 ppm; 152 samples) among items with moderate to high Hg. We also found few studies on freshwater bass from locations considered important for commercial harvest of these fish (e.g., Great Lakes, Canada). However, there are many studies not included in our framework that report Hg values for bass and other freshwater taxa from locations with recreational fisheries (e.g., Lange et al. 1993).

**Figure 3:**
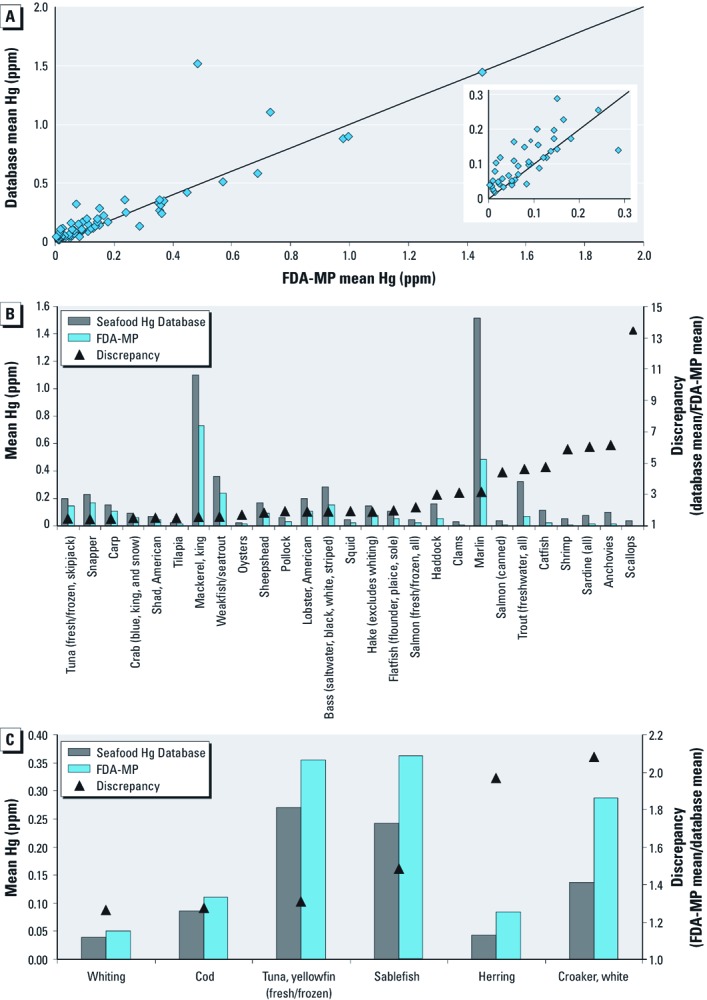
Comparison of Hg content estimated from the Seafood Hg Database (database) with data from the the FDA‑MP. (*A*) Mean Hg content for 58 seafood items relative to the 1:1 line. Inset, mean Hg content estimates for seafood items with mean Hg ≤ 0.3 ppm. (*B*) Seafood items for which FDA‑MP underestimates of mean Hg content are lower than mean estimates based on the Seafood Hg Database (discrepancy = Seafood Hg Database mean/FDA‑MP mean). (*C*) Seafood items for which FDA‑MP overestimates of mean Hg content are higher than the Seafood Hg Database (discrepancy = FDA‑MP mean/ Seafood Hg Database mean; the inverse of discrepancy in (*B*). Larger discrepancy values > 1 indicate larger difference. Seafood items for which discrepancy is < 20% were excluded.

**Figure 4:**
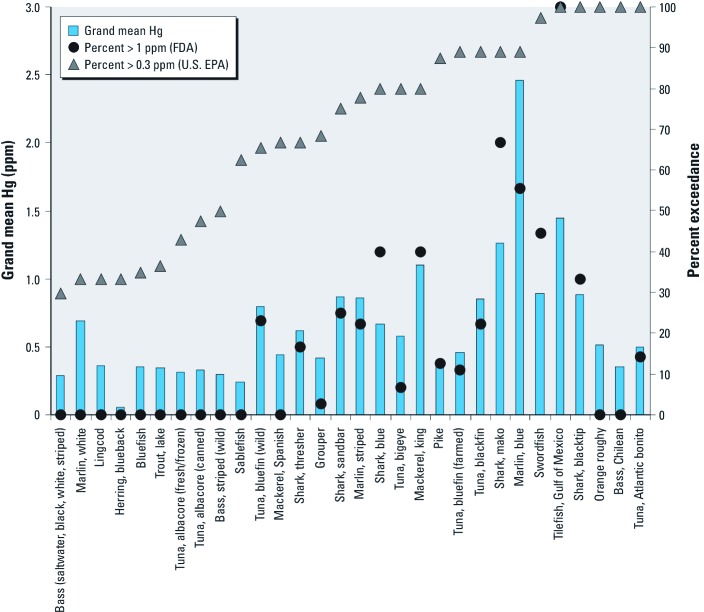
Grand mean Hg concentrations from the Seafood Hg Database and percentage of studies reporting mean Hg concentrations exceeding federal criteria (FDA, 1 ppm; U.S. EPA, 0.3 ppm) by seafood item. Increasing percent of exceedances of U.S. EPA criterion are shown from left to right. Taxa with < 30% exceedances are not included.

*Comparison with FDA-MP and federal criteria.* Mean Hg concentrations from the summarized FDA-MP data (FDA-MP 2011) differed from the grand means estimated from the Seafood Hg Database by ≥ 20% for more than half (33 of 58) of the seafood items listed in the summarized FDA-MP data (Figure 3). Most of these discrepancies were cases in which the FDA-MP estimates for mean Hg content were lower than grand mean estimates from our database (27 of 33 seafood items; Figure 3B). Of these, only marlin, king mackerel, and weakfish/seatrout and freshwater trout were moderate- to high-Hg seafood items. In contrast, FDA-MP estimates of mean Hg content were higher than our grand mean for only 6 seafood items (Figure 3C), all of which were relatively low in Hg. For 30 of the seafood items analyzed and included in the Seafood Hg Database, mean values exceeded the U.S. EPA human health criterion of 0.3 ppm in at least 30% of the observations across studies (Figure 4). In comparison, 6 seafood items exceeded the FDA criterion of 1 ppm in at least 30% of the observations in our database.

## Discussion

Our findings have important implications for estimates of Hg exposure, risk, and the development of seafood consumption advice. First, we found discrepancies in mean Hg content estimated by the FDA-MP (2011) compared with the larger Seafood Hg Database, suggesting that consumption advice and exposure estimates based on the FDA-MP data should be revisited. Most of these discrepancies were cases in which the FDA-MP estimates of seafood Hg content were lower than our estimates. The FDA-MP is a market basket study, whereas our database contains both market basket studies and research studies in which fish were collected directly from their water source. Thus, FDA-MP estimates may be lower than ours because of differences in methodology. However, FDA-MP sampling methods and potential mechanisms resulting in bias relative to the Seafood Hg Database are unclear. Alternatively, FDA-MP estimates may tend to be lower because estimates based on relatively smaller sample sizes are inherently less likely to include rarer high values. In general, although the FDA-MP specifically focuses on Hg concentrations in market seafood that are relevant to typical exposures, Hg estimates based on larger sample sizes are inherently more reliable, particularly given the high degree of Hg variability.

Large discrepancies in estimates of seafood Hg content are likely to result in inaccurate estimates of Hg exposure and risk, particularly for high Hg content seafood items and frequently consumed items. For example, marlin (grand mean Hg, 1.517 ppm; 821 samples) are currently not considered high-Hg fish according to the FDA-MP (mean Hg, 0.485 ppm; 16 samples), even though marlin have Hg concentrations similar to those of shark, swordfish, and tilefish from the Gulf of Mexico, for which consumption limits are recommended to reduce risky Hg exposure. Most of the discrepancies for which the FDA-MP’s estimates of Hg content are lower than ours are for low-Hg seafood and are likely to have minor health consequences compared with discrepancies of moderate- to high-Hg seafood. However, many of these low-Hg seafood items (e.g., shrimp, clams, flounder) are among the most popular with U.S. consumers (Groth 2010). Hence, consumption of these items may result in Hg exposures that exceed previous estimates for the U.S. population. In addition, our results suggest that certain seafood items, such as yellowfin tuna (grand mean Hg, 0.270 ppm; 1,183 samples), contain lower Hg concentrations than estimated by the FDA-MP (mean Hg, 0.354 ppm; 231 samples) and that increased consumption of these items may be possible with negligible risk. Our analyses of the percentage of Hg values that exceed federal criteria provide further insight into the seafood items that should be the focus of management and policy development.

Finally, we found higher variability in seafood Hg concentrations than previously observed (Sunderland 2007). This high variability reflects the framework of the Seafood Hg Database, which encompasses variability across geographic regions, time, fish size class, and other factors that vary within the overall U.S. market but are typically constrained within individual studies. Together, the discrepancies and high variability of seafood Hg concentrations we observed based on a large aggregation of data indicate that smaller data sets are more susceptible to random sampling error and may be inadequate aids to developing public health policy or scientific understanding. Although smaller individual data sets may be more accurate for estimating exposures in specific local populations, they may not reflect the full range of seafood Hg concentrations in the U.S. market.

There is a clear need to identify and compare the key sources of variability in seafood Hg content, and to translate this information into consumption advice and exposure and risk analyses. Many studies of freshwater fish have identified factors that influence Hg variability. These factors, including physicochemical (pH, dissolved organic carbon, nutrient availability) (Chen et al. 2005; Driscoll et al. 1995) and ecophysiological factors (food chain length, body size) (Borgmann and Whittle 1991; Cabana et al. 1994; Chen et al. 2000) are often confounded and vary among ecosystems and over time. Compared with the freshwater literature, fewer studies have examined links between Hg content of seafood and factors such as body size and geographic harvest region (Sunderland 2007). Future efforts should account for and identify the key factors influencing Hg content in commercial seafood (e.g., body size, trophic level) as well as compare differences in Hg content among geographic regions. Progress is more likely if large monitoring studies explicitly report data on these factors together with seafood Hg data. Research efforts examining the influence of these factors in commercial fish and shellfish are critical to better predict changes in Hg content of commercial seafood.

Our analyses highlight challenges associated with characterizing variability of seafood Hg across studies as well as potential sources of bias. Accurate assessments of exposure and risk are ideally derived using probability distributions based on raw data (Sioen et al. 2007; World Health Organization 2000). However, many of the studies that we reviewed, particularly from the academic literature, did not report raw values and less than half of all studies reported SDs or SEs. To capitalize on the abundance of aggregate data in the literature (e.g., mean values), additional studies should test and validate methods used to generate probability distributions (World Health Organization 2000). Our estimates of variability of seafood Hg content are likely to be influenced by the types of available data. For example, differences in data collection methods among studies, such as analysis of fillet versus whole fish, reporting MeHg instead of total Hg values, including samples with concentrations below detection limits, and noting differences in fish size (often not reported), are likely to introduce variability in overall Hg estimates. Moreover, geographic and temporal factors, both within and between studies, may contribute to our estimates of variability. Standardization or consistent disclosure of measurement methods would greatly facilitate comparison and aggregation of data into larger data sets that can be used to monitor exposure and risk.

Our results demonstrate that lower Hg concentrations in farmed fish compared with wild fish is broadly consistent, despite high variability typical of fish Hg concentrations across studies, for each seafood item analyzed. However, Hg data for farmed fish are relatively scarce. Thus, there is a need for more extensive study of Hg concentration patterns in farmed versus wild fish, as well as for the factors that influence them. Nevertheless, given the increase in global consumption of farm-raised fish (NMFS 2010), their Hg levels should be distinguished from those of wild fish and explicitly incorporated into consumption advice and risk analyses.

Although previous studies have shown lower Hg levels in farmed fish than in wild fish, they have typically focused on individual taxa (Balshaw et al. 2008; Dasgupta et al. 2004), primarily salmon (Easton et al. 2002; Foran et al. 2004), and on fish from only a few sources (Dasgupta et al. 2004; Easton et al. 2002). Moreover, the pattern is not universal. At least three studies found no difference in Hg levels between farmed and wild salmon (Easton et al. 2002; Foran et al. 2004) and farmed and wild cod (Jardine et al. 2009). In contrast, our study found consistently lower mean Hg concentrations in farmed seafood across studies for multiple seafood items. In some cases, differences in Hg content between farmed and wild seafood may partly reflect taxonomic differences. For example, farmed trout (mostly rainbow trout) have Hg concentrations similar to those of wild rainbow trout but lower than in wild lake trout. Lower Hg in farmed fish also may be due to ecological characteristics unique to aquaculture settings, such as lower Hg levels in feed, shorter food chain lengths, or a growth dilution effect via higher growth efficiency (Karimi et al. 2010). More broadly, our findings contrast with studies that have found higher concentrations of persistent organic pollutants (e.g., polychlorinated biphenyls, dioxins, and pesticides) in certain types of farmed fish (Hites et al. 2004; Kelly et al. 2011), possibly reflecting the content of the diet provided in aquaculture operations. Therefore, understanding the mechanisms behind differences in contaminant content in farmed and wild seafood is a necessary step toward effectively managing production of farmed seafood.

Our analyses support the need to revise monitoring efforts of both seafood Hg content and characteristics of the U.S. seafood market in order to better track human exposure and potential health risk. In general, to better understand seafood Hg concentrations, monitoring efforts should focus on seafood items that tend to exceed federal criteria (e.g., the U.S. EPA criterion of 0.3 ppm), that are relatively understudied, or that have highly variable Hg content. Specifically, our results suggest a need to increase monitoring of imported seafood from Asia and South America, farmed seafood, and specific seafood items that have been understudied. Increased monitoring efforts may be particularly important for understudied high-Hg seafood items. For example, tilefish is thought to pose a high risk of MeHg exposure (FDA 2004) because of estimates of Hg content for tilefish collected from the Gulf of Mexico in the 1970s (FDA-MP 2011; Hall et al. 1978). Current estimates of tilefish collected from a more geographically extensive region are needed to test whether tilefish continue to pose a health risk. In addition, improved traceability and transparency of the U.S. seafood market is critical to control Hg exposure and risk by providing information about seafood sources (e.g., country of origin) and taxonomic identity. Complex market linkages, including reexports of imported fish, change over time and are largely unaccounted for in market data (e.g., NMFS 2010), yet are necessary to track exposure from geographic origin of fish to consumers. Increasing imports, together with a lack of market traceability (Jacquet and Pauly 2008) and seafood identification practices (Lowenstein et al. 2009), challenge our ability to estimate exposure, because both geographic origin (Sunderland 2007) and species identity are important determinants of seafood Hg content. Ideal monitoring efforts will need to consider changes in market sources, species composition and size, along with human consumption patterns.

## Conclusions

Our findings suggest that seafood consumption advice and exposure estimates based on smaller data sets, such as the FDA-MP, should be revisited using larger data sets that are more likely to capture accurate estimates of mean Hg values and their variability in U.S. commercial seafood. Priorities for new research should include increased monitoring of farmed seafood and imported seafood from Asia and South America, as well as studies examining the processes underlying lower Hg concentrations in farmed seafood. Finally, additional studies should compare the relative influence of different environmental and ecological factors on the variability of seafood Hg content.

## Supplemental Material

(475 KB) PDF

(229 KB) XLS
