# Supplementary material for: A Quantitative Synthesis of Mercury in Commercial Seafood and Implications for Exposure in the United States
Source: Environ Health Perspect. 2012 Jun 25;120(11):1512–9. doi: 10.1289/ehp.1205122 (PMC3556626; doi:10.1289/ehp.1205122)
Supplement: (475 KB) PDF [file ehp.1205122.s001.pdf]

# Supplemental Material

# A Quantitative Synthesis of Mercury in Commercial Seafood and Implications for Exposure in the U.S.

**Roxanne Karimi, Timothy P. Fitzgerald, Nicholas S. Fisher**

## Table of Contents

Supplemental Material, Table S1: Summary of Hg concentrations across studies in commonly consumed seafood items in the U.S. .......... **2**

Supplemental Material, Table S2: Seafood Hg Database ............(provided as a separate excel file)

Search Terms for Supplemental Material, Table S2: Seafood Hg Database .......... **5**

References for Supplemental Material, Table S2: Seafood Hg Database .......... **6**

Table S1. (continues through page 4)

**Supplemental Material, Table S1. Summary of Hg concentrations across studies in commonly consumed seafood items in the U.S.**

| *Seafood Item* | *Grand Mean Hg (ppm)*[a] | *Samples (Total)*[b] | *$SE_W$* | *$SD_W$* | *Min, Max (ppm)*[c] | *CV* |
|---|---|---|---|---|---|---|
| Anchovies (All) | 0.103 | 455 | 0.041 | 0.197 | (0.008, 0.154) | 1.91 |
| Bass (Chilean) | 0.357 | 100 | 0.041 | 0.185 | (0.310, 0.600) | 0.52 |
| Bass (Freshwater, All) | 0.170 | 149 | 0.059 | 0.361 | (0.118, 0.242) | 2.12 |
| Bass (Saltwater, Black, White, Striped) | 0.288 | 1660 | 0.150 | 1.004 | (0.005, 0.650) | 3.49 |
| Bass, Striped (All) | 0.285 | 1367 | 0.140 | 1.155 | (0.028, 0.650) | 4.05 |
| Bass, Striped (farmed) | 0.028 | 15 | NA | NA | (0.028, 0.028) | NA |
| Bass, Striped (wild) | 0.295 | 1311 | 0.134 | 1.147 | (0.094, 0.650) | 3.89 |
| Bluefish | 0.351 | 1019 | 0.145 | 0.965 | (0.034, 0.680) | 2.75 |
| Butterfish | 0.054 | 109 | 0.021 | 0.112 | (0.004, 0.080) | 2.08 |
| Carp (All) | 0.156 | 477 | 0.095 | 0.521 | (0.030, 0.472) | 3.35 |
| Catfish (All) | 0.118 | 1757 | 0.087 | 0.586 | (0.005, 0.714) | 4.97 |
| Catfish (wild, all species) | 0.144 | 1396 | 0.078 | 0.513 | (0.005, 0.714) | 3.57 |
| Catfish, Channel (wild) | 0.120 | 521 | 0.038 | 0.253 | (0.020, 0.231) | 2.10 |
| Catfish (farmed, all species) | 0.012 | 320 | 0.008 | 0.073 | (0.008, 0.030) | 6.14 |
| Clams (All) | 0.028 | 1027 | 0.032 | 0.177 | (0.005, 0.300) | 6.29 |
| Clams, Hard | 0.047 | 181 | 0.026 | 0.130 | (0.005, 0.300) | 2.75 |
| Clams, Geoduck | 0.030 | 11 | 0.021 | 0.049 | (0.010, 0.041) | 1.66 |
| Clams, Cockle | 0.054 | 122 | 0.073 | 0.404 | (0.019, 0.253) | 7.47 |
| Clams, Pacific Littleneck | 0.022 | 18 | 0.009 | 0.022 | (0.011, 0.028) | 0.99 |
| Clams, Softshell | 0.016 | 471 | 0.020 | 0.249 | (0.008, 0.086) | 15.42 |
| Cod (All) | 0.087 | 2115 | 0.038 | 0.358 | (0.019, 0.176) | 4.12 |
| Cod, Atlantic (farmed) | 0.034 | 24 | NA | NA | (0.034, 0.034) | NA |
| Cod, Atlantic (wild) | 0.070 | 1452 | 0.017 | 0.261 | (0.035, 0.113) | 3.70 |
| Cod, Pacific | 0.144 | 431 | 0.038 | 0.260 | (0.019, 0.176) | 1.81 |
| Crab (All) | 0.098 | 1564 | 0.086 | 0.453 | (0.005, 0.302) | 4.61 |
| Crab (Blue, King and Snow) | 0.095 | 1087 | 0.098 | 0.526 | (0.005, 0.302) | 5.56 |
| Crab, Blue | 0.110 | 864 | 0.103 | 0.594 | (0.014, 0.302) | 5.40 |
| Crab, Dungeness | 0.120 | 264 | 0.037 | 0.225 | (0.051, 0.192) | 1.88 |
| Crab, King | 0.027 | 203 | 0.032 | 0.154 | (0.005, 0.101) | 5.63 |
| Crab, Snow | 0.110 | 20 | 0.073 | 0.187 | (0.048, 0.223) | 1.71 |
| Crawfish (All) | 0.034 | 206 | 0.019 | 0.104 | (0.021, 0.210) | 3.03 |
| Croaker (All) | 0.092 | 856 | 0.058 | 0.308 | (0.011, 0.287) | 3.36 |
| Croaker, Atlantic | 0.069 | 572 | 0.025 | 0.135 | (0.011, 0.146) | 1.97 |
| Croaker, White | 0.169 | 193 | 0.066 | 0.344 | (0.040, 0.287) | 2.04 |
| Cuttlefish | 0.134 | 156 | 0.085 | 0.275 | (0.020, 0.270) | 2.05 |
| Eel (All) | 0.186 | 986 | 0.111 | 0.608 | (0.030, 0.800) | 3.28 |
| Eel (wild) | 0.216 | 659 | 0.110 | 0.551 | (0.030, 0.800) | 2.55 |
| Eel (farmed) | 0.066 | 220 | 0.027 | 0.163 | (0.049, 0.181) | 2.46 |
| Flatfish (Flounder, Plaice, Sole) | 0.110 | 3070 | 0.079 | 0.417 | (0.005, 0.463) | 3.80 |
| Flounder (All) | 0.119 | 1687 | 0.075 | 0.406 | (0.005, 0.463) | 3.42 |
| Flounder, Summer | 0.121 | 427 | 0.042 | 0.216 | (0.005, 0.184) | 1.79 |
| Flounder, Windowpane | 0.152 | 84 | 0.037 | 0.152 | (0.105, 0.180) | 1.00 |
| Flounder, Winter | 0.070 | 302 | 0.039 | 0.228 | (0.021. 0.146) | 3.27 |
| Freshwater Perch (All) | 0.141 | 1295 | 0.110 | 0.745 | (0.014, 0.810) | 5.27 |
| Grouper (All) | 0.417 | 643 | 0.196 | 0.804 | (0.035, 1.080) | 1.93 |
| Haddock (All) | 0.164 | 226 | 0.166 | 0.752 | (0.020, 0.381) | 4.59 |
| Hake (All) | 0.146 | 739 | 0.090 | 0.489 | (0.015, 0.269) | 3.36 |
| Halibut (All) | 0.254 | 3532 | 0.060 | 0.703 | (0.036, 0.450) | 2.76 |
| Halibut, Pacific | 0.261 | 3111 | 0.053 | 1.127 | (0.158, 0.450) | 4.32 |

Table S1. (continues through page 4)

| Seafood Item | Grand Mean Hg (ppm)[a] | Samples (Total)[b] | $SE_W$ | $SD_W$ | Min, Max (ppm)[c] | CV |
|---|---|---|---|---|---|---|
| Halibut, Greenland | 0.183 | 138 | 0.120 | 0.630 | (0.040, 0.289) | 3.44 |
| Herring (All) | 0.043 | 1277 | 0.026 | 0.174 | (0.010, 0.321) | 4.09 |
| Herring, Atlantic | 0.037 | 973 | 0.015 | 0.119 | (0.010, 0.132) | 3.19 |
| Herring, Pacific | 0.060 | 194 | 0.048 | 0.300 | (0.017, 0.140) | 4.97 |
| Lingcod | 0.363 | 333 | 0.128 | 0.952 | (0.080, 0.440) | 2.62 |
| Lobster (All) | 0.153 | 344 | 0.070 | 0.315 | (0.042, 0.249) | 2.06 |
| Lobster, American | 0.200 | 142 | 0.075 | 0.367 | (0.045, 0.249) | 1.84 |
| Lobster, Spiny | 0.100 | 62 | 0.035 | 0.137 | (0.064, 0.210) | 1.36 |
| Mackerel (All) | 0.586 | 2481 | 0.450 | 3.237 | (0.008, 1.510) | 5.53 |
| Mackerel, Atlantic | 0.045 | 191 | 0.037 | 0.192 | (0.033, 0.270) | 4.30 |
| Mackerel, Chub | 0.099 | 129 | 0.033 | 0.166 | (0.028, 0.152) | 1.68 |
| Mackerel, King | 1.101 | 821 | 0.383 | 3.470 | (0.110, 1.510) | 3.15 |
| Mackerel, Spanish | 0.440 | 1168 | 0.097 | 1.105 | (0.147, 0.530) | 2.51 |
| Marlin (All) | 1.517 | 821 | 1.654 | 7.495 | (0.140, 10.520) | 4.94 |
| Marlin, Blue | 2.465 | 364 | 2.120 | 9.532 | (0.190, 10.520) | 3.87 |
| Marlin, Striped | 0.861 | 179 | 0.528 | 2.356 | (0.140, 1.720) | 2.74 |
| Marlin, White | 0.695 | 56 | 0.120 | 0.518 | (0.270, 0.718) | 0.75 |
| Monkfish | 0.174 | 92 | 0.024 | 0.117 | (0.083, 0.198) | 0.67 |
| Mullet | 0.050 | 638 | 0.027 | 0.152 | (0.006, 0.310) | 3.05 |
| Mussels (All) | 0.028 | 755 | 0.016 | 0.106 | (0.013, 0.085) | 3.84 |
| Ocean Perch | 0.117 | 262 | 0.082 | 0.421 | (0.010, 0.548) | 3.59 |
| Orange Roughy | 0.513 | 152 | 0.103 | 0.569 | (0.350, 0.595) | 1.11 |
| Oysters (All) | 0.020 | 5310 | 0.013 | 0.178 | (0.005, 0.083) | 9.00 |
| Oysters, Eastern | 0.018 | 4573 | 0.009 | 0.161 | (0.006, 0.083) | 8.87 |
| Oysters, Pacific | 0.039 | 290 | 0.025 | 0.171 | (0.008, 0.070) | 4.37 |
| Pike | 0.404 | 1374 | 0.101 | 1.328 | (0.247, 1.340) | 3.29 |
| Plaice | 0.148 | 282 | 0.137 | 0.576 | (0.015, 0.343) | 3.88 |
| Pollock (All) | 0.058 | 540 | 0.059 | 0.342 | (0.005, 0.704) | 5.93 |
| Pollock, Atlantic | 0.160 | 79 | 0.053 | 0.330 | (0.030, 0.170) | 2.07 |
| Pollock, Pacific/Alaska | 0.050 | 235 | 0.027 | 0.145 | (0.005, 0.140) | 2.89 |
| Porgy | 0.065 | 169 | 0.027 | 0.143 | (0.033, 0.102) | 2.20 |
| Sablefish | 0.243 | 477 | 0.080 | 0.620 | (0.151, 0.517) | 2.55 |
| Salmon (All) | 0.048 | 2818 | 0.023 | 0.143 | (0.005, 0.190) | 3.00 |
| Salmon, Atlantic (farmed) | 0.026 | 145 | 0.020 | 0.077 | (0.005, 0.117) | 2.91 |
| Salmon, Atlantic (wild) | 0.058 | 95 | 0.015 | 0.083 | (0.047, 0.073) | 1.43 |
| Salmon, Chinook, farmed | 0.017 | 4 | 0.017 | 0.024 | (0.005, 0.029) | 1.41 |
| Salmon, Chinook, wild | 0.067 | 580 | 0.013 | 0.106 | (0.041, 0.090) | 1.59 |
| Salmon, Chum | 0.046 | 456 | 0.018 | 0.139 | (0.014, 0.082) | 3.00 |
| Salmon, Coho | 0.044 | 567 | 0.007 | 0.065 | (0.016, 0.063) | 1.49 |
| Salmon, Pink | 0.037 | 222 | 0.009 | 0.064 | (0.005, 0.040) | 1.72 |
| Salmon, Sockeye | 0.039 | 396 | 0.004 | 0.026 | (0.005, 0.057) | 0.66 |
| Salmon (canned) | 0.035 | 61 | 0.012 | 0.042 | (0.028, 0.090) | 1.20 |
| Sardine (All) | 0.079 | 1007 | 0.036 | 0.201 | (0.010, 0.330) | 2.56 |
| Scallops (All) | 0.040 | 336 | 0.033 | 0.148 | (0.004, 0.090) | 3.65 |
| Seabass, Black | 0.120 | 139 | 0.032 | 0.118 | (0.005, 0.150) | 0.98 |
| Shad (All) | 0.077 | 93 | 0.031 | 0.099 | (0.040, 0.156) | 1.29 |
| Shad, American | 0.067 | 76 | 0.019 | 0.095 | (0.040, 0.111) | 1.42 |
| Shark (All) | 0.882 | 3722 | 0.462 | 2.504 | (0.080, 8.250) | 2.84 |
| Shark, Blacktip | 0.882 | 250 | 0.274 | 1.249 | (0.380, 2.140) | 1.42 |
| Shark, Blue | 0.664 | 50 | 0.480 | 1.516 | (0.270, 1.900) | 2.28 |
| Shark, Mako | 1.259 | 166 | 0.464 | 1.995 | (0.206, 3.200) | 1.58 |
| Shark, Sandbar | 0.869 | 115 | 0.301 | 1.141 | (0.082, 1.397) | 1.31 |
| Shark, Thresher | 0.622 | 119 | 0.421 | 1.874 | (0.130, 1.300) | 3.01 |

Table S1. (continues through page 4)

| ***Seafood Item*** | ***Grand Mean Hg (ppm)***[a] | ***Samples (Total)***[b] | $SE_W$ | $SD_W$ | ***Min, Max (ppm)***[c] | ***CV*** |
|---|---|---|---|---|---|---|
| Sheepshead | 0.166 | 340 | 0.038 | 0.347 | (0.128, 0.197) | 2.09 |
| Shrimp (All) | 0.053 | 935 | 0.053 | 0.212 | (0.003, 0.380) | 4.03 |
| Shrimp, Brown | 0.077 | 72 | 0.024 | 0.083 | (0.020, 0.130) | 1.07 |
| Shrimp, Pink | 0.083 | 49 | 0.016 | 0.079 | (0.005, 0.085) | 0.95 |
| Shrimp, White | 0.057 | 113 | 0.036 | 0.136 | (0.006, 0.200) | 2.38 |
| Skate (All) | 0.138 | 70 | 0.027 | 0.093 | (0.032, 0.214) | 0.67 |
| Smelt | 0.025 | 175 | 0.019 | 0.086 | (0.008, 0.125) | 4.03 |
| Snapper (All) | 0.230 | 1244 | 0.104 | 0.514 | (0.031, 0.776) | 2.27 |
| Snapper, Gray | 0.233 | 699 | 0.068 | 0.595 | (0.121, 0.348) | 2.55 |
| Snapper, Red | 0.243 | 279 | 0.168 | 0.725 | (0.031, 0.776) | 2.98 |
| Sole | 0.086 | 1101 | 0.056 | 0.310 | (0.013, 0.308) | 3.62 |
| Squid | 0.044 | 728 | 0.024 | 0.130 | (0.008, 0.095) | 2.99 |
| Swordfish | 0.893 | 1726 | 0.296 | 2.052 | (0.150, 3.310) | 2.30 |
| Tilapia | 0.019 | 129 | 0.027 | 0.097 | (0.002, 0.150) | 4.99 |
| Tilefish (All) | 0.883 | 109 | 0.695 | 2.962 | (0.080, 1.450) | 3.35 |
| Tilefish, Atlantic | 0.171 | 47 | 0.049 | 0.195 | (0.131, 0.229) | 1.14 |
| Tilefish, Gulf of Mexico | 1.445 | 61 | 0.059 | 0.324 | (1.123, 1.450) | 0.22 |
| Trout (freshwater, wild and unknown status) | 0.344 | 2804 | 0.087 | 1.030 | (0.030, 0.440) | 3.00 |
| Trout, Lake | 0.349 | 2748 | 0.080 | 1.268 | (0.047, 0.440) | 3.63 |
| Trout (freshwater, farmed) | 0.029 | 178 | 0.015 | 0.066 | (0.005, 0.060) | 2.30 |
| Tuna (fresh/frozen, All) | 0.450 | 3780 | 0.340 | 1.619 | (0.007, 3.030) | 3.59 |
| Tuna, Albacore | 0.317 | 296 | 0.103 | 0.475 | (0.030, 0.500) | 1.50 |
| Tuna, Atlantic bonito | 0.499 | 263 | 0.359 | 2.200 | (0.326, 1.662) | 4.41 |
| Tuna, Bigeye | 0.582 | 376 | 0.222 | 1.113 | (0.114, 1.149) | 1.91 |
| Tuna, Blackfin | 0.856 | 159 | 0.231 | 0.972 | (0.200, 1.160) | 1.14 |
| Tuna, Bluefin (farmed) | 0.455 | 108 | 0.156 | 0.540 | (0.190, 1.020) | 1.19 |
| Tuna, Bluefin (wild) | 0.796 | 514 | 0.542 | 2.408 | (0.057, 3.030) | 3.03 |
| Tuna, Skipjack | 0.198 | 341 | 0.083 | 0.320 | (0.060, 0.450) | 1.62 |
| Tuna, Yellowfin | 0.270 | 1183 | 0.125 | 0.797 | (0.030, 0.650) | 2.95 |
| Tuna, Albacore (canned) | 0.328 | 1362 | 0.113 | 0.955 | (0.155, 0.588) | 2.92 |
| Tuna, Light (canned or packed) | 0.118 | 972 | 0.038 | 0.300 | (0.047, 0.400) | 2.55 |
| Tuna, Yellowfin (canned) | 0.143 | 298 | 0.098 | 0.688 | (0.029, 0.240) | 4.80 |
| Weakfish/Seatrout (All) | 0.361 | 2105 | 0.193 | 1.348 | (0.021, 1.060) | 3.73 |
| Whitefish (All) | 0.106 | 2721 | 0.051 | 0.707 | (0.018, 0.352) | 6.70 |
| Whiting | 0.040 | 27 | 0.015 | 0.056 | (0.030, 0.051) | 1.38 |

$SE_w$ = Weighted standard error (see main text for details)

$SD_w$ = Weighted standard deviation (see main text for details)

CV = Coefficient of variation

[a]Grand Mean Hg calculated across studies, based on wet weight

[b]Total samples across studies

[c]Minimum and maximum mean reported across studies

## Search Terms for Supplemental Material, Table S2: Seafood Hg Database

mercury and  anchovies
mercury and anchovy
mercury and bass
mercury and bluefish
mercury and buffalofish
mercury and butterfish
mercury and carp
mercury and catfish
mercury and clam
mercury and cod
mercury and crab
mercury and crayfish
mercury and croaker
mercury and flounder
mercury and grouper
mercury and haddock
mercury and hake
mercury and halibut
mercury and herring
mercury and lincod
mercury and lobster
mercury and mackerel
mercury and marlin
mercury and monkfish
mercury and mullet
mercury and mussel
mercury and orange roughy
mercury and oyster
mercury and perch
mercury and pike
mercury and plaice
mercury and pollock
mercury and porgy
mercury and sablefish
mercury and salmon
mercury and sardine
mercury and scallop
mercury and scorpionfish
mercury and sea trout
mercury and seatrout
mercury and shad
mercury and shark
mercury and sheepshead
mercury and shrimp
mercury and skate
mercury and smelt
mercury and snapper
mercury and sole
mercury and squid
mercury and swordfish
mercury and tilapia
mercury and tilefish
mercury and trout
mercury and tuna
mercury and whitefish

**References for Supplemental Material, Table 2: Seafood Hg Database**


Adams D, Sonneb C, Basuc N, Dietzb R, Namc D, Leifssond P, et al. 2010. Mercury contamination in spotted seatrout, *Cynoscion nebulosus*: An assessment of liver, kidney, blood, and nervous system health. Sci Total Environ 408(23):5808-5816.

Adams DH. 2004. Total mercury levels in tunas from offshore waters of the Florida Atlantic coast. Mar Pollut Bull 49(7-8):659-663.

Adams DH, McMichael RH. 1999. Mercury levels in four species of sharks from the Atlantic coast of Florida. Fishery Bulletin 97(2):372-379.

Adams DH, McMichael RH. 2007. Mercury in king mackerel, *Scomberomorus cavalla*, and Spanish mackerel, S-maculatus, from waters of the south-eastern USA: regional and historical trends. Mar Freshw Res 58(2):187-193.

Adams DH, McMichael RH, Henderson GE. 2003. Florida Marine Research Institute Technical Report TR-9, Mercury Levels in Marine and Estuarine Fishes of Florida 1989-2001.

Africa CR, Pascual AE, Santiago EC. 2009. Total Mercury in Three Fish Species Sold in a Metro Manila Public Market: Monitoring and Health Risk Assessment. Science Diliman 21(1):1-6.

Amlund H, Lundebye AK, Berntssen MHG. 2007. Accumulation and elimination of methylmercury in Atlantic cod (*Gadus morhua* L.) following dietary exposure. Aquat Toxicol 83(4):323-330.

Andersen JL, Depledge MH. 1997. A survey of total mercury and methylmercury in edible fish and invertebrates from Azorean waters. Mar Environ Res 44(3):331-350.

Arcos JM, Ruiz X, Bearhop S, Furness RW. 2002. Mercury levels in seabirds and their fish prey at the Ebro Delta (NW Mediterranean): the role of trawler discards as a source of contamination. Mar Ecol-Prog Ser 232:281-290.

Ashraf W. 2006. Levels of selected heavy metals in tuna fish. Arab J Sci Eng 31(1A):89-92.

Augelli MA, Munoz RAA, Richter EM, Cantagallo MI, Angnes L. 2007. Analytical procedure for total mercury determination in fishes and shrimps by chronopotentiometric stripping analysis at gold film electrodes after microwave digestion. Food Chem 101(2):579-584.

Baeyens W, Leermakers M, Papina T, Saprykin A, Brion N, Noyen J, et al. 2003. Bioconcentration and biomagnification of mercury and methylmercury in North Sea and Scheldt estuary fish. Arch Environ Contam Toxicol 45(4):498-508.

Bahnick D, Sauer C, Butterworth B, Kuehl DW. 1994. A National Study of Mercury Contamination of Fish. 4. Analytical Methods and Results. Chemosphere 29(3):537-546.

Balshaw S, Edwards JW, Ross KE, Ellis D, Padula DJ, Daughtry BJ. 2008. Empirical models to identify mechanisms driving reductions in tissue mercury concentration during culture of farmed southern bluefin tuna *Thunnnus maccoyii*. Mar Pollut Bull 56(12):2009-2017.

Bank MS, Chesney E, Shine JP, Maage A, Senn DB. 2007. Mercury bioaccumulation and trophic transfer in sympatric snapper species from the Gulf of Mexico. Ecological Applications 17:2100-2110.

Barska I, Skrzynski I. 2003. Contents of methylmercury and total mercury in Baltic Sea fish and fish products. Bulletin of the Sea Fisheries Institute 3(160):3-15.

Berntssen MHG, Julshamn K, Lundebye AK. 2010. Chemical contaminants in aquafeeds and Atlantic salmon (*Salmo salar*) following the use of traditional- versus alternative feed ingredients. Chemosphere 78(6):637-646.

Bethune C, Seierstad SL, Seljeflot I, Johansen O, Arnesen H, Meltzer HM, et al. 2006. Dietary intake of differently fed salmon: a preliminary study on contaminants. European Journal of Clinical Investigation 36(3):193-201.

Blanco S, Gonzalez JC, Vieites JM. 2008. Mercury, cadmium and lead levels in samples of the main traded fish and shellfish species in Galicia, Spain. Food Addit Contam Part B-Surveill 1(1):15-21.

Bloom NS. 1992. On the chemical form of mercury in edible fish and marine invertebrate tissue Can J Fish Aquat Sci 49(5):1010-1017.

Bordajandi LR, Gomez G, Abad E, Rivera J, Fernandez-Baston MD, Blasco J, et al. 2004. Survey of persistent organochlorine contaminants (PCBs, PCDD/Fs, and PAHs), heavy metals (Cu, Cd, Zn,

Pb, and Hg), and arsenic in food samples from Huelva (Spain): Levels and health implications. J Agric Food Chem 52(4):992-1001.

Boscher A, Gobert S, Guignard C, Ziebel J, L'Hoste L, Gutleb AC, et al. 2010. Chemical contaminants in fish species from rivers in the North of Luxembourg: Potential impact on the Eurasian otter (*Lutra lutra*). Chemosphere 78(7):785-792.

Braune B, Muir D, DeMarch B, Gamberg M, Poole K, Currie R, et al. 1999. Spatial and temporal trends of contaminants in Canadian Arctic freshwater and terrestrial ecosystems: a review. Sci Total Environ 230(1-3):145-207.

Braune BM. 1987. Mercury accumulation in relation to size and age of atlantic herring (*Clupea harengus harengus*) from the southwestern Bay of Fundy, Canada. Arch Environ Contam Toxicol 16(3):311-320.

Brinkmann L, Rasmussen JB. 2010. High levels of mercury in biota of a new Prairie irrigation reservoir with a simplified food web in Southern Alberta, Canada. Hydrobiologia 641(1):11-21.

Brockman JD, Sharp N, Ngwenyama RA, Shelnutt LD, McElroy JA. 2009. The concentration and variability of selenium and mercury measured in vacuum-packed tuna fish. J Radioanal Nucl Chem 282(1):45-48.

Burger J. 2009. Risk to consumers from mercury in bluefish (*Pomatomus saltatrix*) from New Jersey: Size, season and geographical effects. Environ Res 109(7):803-811.

Burger J, Gochfeld M. 2004. Mercury in canned tuna: white versus light and temporal variation. Environ Res 96(3):239-249.

Burger J, Gochfeld M. 2005. Heavy metals in commercial fish in New Jersey. Environ Res 99(3):403-412.

Burger J, Gochfeld M. 2006. Mercury in fish available in supermarkets in Illinois: Are there regional differences. Sci Total Environ 367(2-3):1010-1016.

Burger J, Gochfeld M, Jeitner C, Burke S, Stamm T. 2007c. Metal levels in flathead sole (*Hippoglossoides elassodon*) and great sculpin (*Myoxocephalus polyacanthocephalus*) from Adak Island, Alaska: Potential risk to predators and fishermen. Environ Res 103(1):62-69.

Burger J, Jeitner C, Donio M, Shukla S, Gochfeld M. 2009. Factors Affecting Mercury and Selenium Levels in New Jersey Flatfish: Low Risk to Human Consumers. J Toxicol Env Health Part A 72(14):853-860.

Burger J, Gaines KF, Boring CS, Stephens WL, Snodgrass J, Gochfeld M. 2001. Mercury and selenium in fish from the Savannah River: Species, trophic level, and locational differences. Environ Res 87(2):108-118.

Burger J, Gochfeld M, Jeitner C, Burke S, Stamm T, Snigaroff R, et al. 2007b. Mercury levels and potential risk from subsistence foods from the Aleutians. Sci Total Environ 384(1-3):93-105.

Burger J, Gaines KF, Boring CS, Stephens WL, Snodgrass J, Dixon C, et al. 2002. Metal levels in fish from the Savannah River: Potential hazards to fish and other receptors. Environ Res 89(1):85-97.

Burger J, Gochfeld M, Shukla T, Jeitner C, Burke S, Donio M, et al. 2007a. Heavy metals in Pacific Cod (*Gadus macrocephalus*) from the Aleutians: Location, age, size, and risk. J Toxicol Env Health Part A 70(22):1897-1911.

Bustamante P, Lahaye V, Durnez C, Churlaud C, Caurant F. 2006. Total and organic Hg concentrations in cephalopods from the North Eastern Atlantic waters: Influence of geographical origin and feeding ecology. Sci Total Environ 368(2-3):585-596.

Butala SJM, Scanlan LP, Chaudhur SN, Perry DD, Taylor R. 2007. Interlaboratory bias in the determination of mercury concentrations in commercially available fish utilizing thermal decomposition/amalgamation atomic absorption spectrophotometry. J Food Prot 70(10):2422-2425.

Cabanero AI, Madrid Y, Camara C. 2004. Selenium and mercury bioaccessibility in fish samples: an in vitro digestion method. Anal Chim Acta 526(1):51-61.

Cabanero AI, Madrid Y, Camara C. 2007. Mercury-selenium species ratio in representative fish samples and their bioaccessibility by an in vitro digestion method. Biol Trace Elem Res 119(3):195-211.

Cabanero AI, Carvalho C, Madrid Y, Batoreu C, Camara C. 2005. Quantification and speciation of mercury and selenium in fish samples of high consumption in Spain and Portugal. Biol Trace Elem Res 103(1):17-35.
Cai Y, Rooker JR, Gill GA, Turner JP. 2007. Bioaccumulation of mercury in pelagic fishes from the northern Gulf of Mexico. Can J Fish Aquat Sci 64(3):458-469.
Caldwell RS, Buhler DR. 1983. Heavy metals in estuarine shellfish from Oregon Arch Environ Contam Toxicol 12(1):15-23.
Campbell L, Hecky RE, Dixon DG, Chapman LJ. 2006. Food web structure and mercury transfer in two contrasting Ugandan highland crater lakes (East Africa). Afr J Ecol 44(3):337-346.
Campbell LM, Osano O, Hecky RE, Dixon DG. 2003b. Mercury in fish from three rift valley lakes (Turkana, Naivasha and Baringo), Kenya, East Africa. Environ Pollut 125(2):281-286.
Campbell LM, Hecky RE, Nyaundi J, Muggide R, Dixon DG. 2003a. Distribution and food-web transfer of mercury in Napoleon and Winam Gulfs, Lake Victoria, East Africa. J Gt Lakes Res 29:267-282.
Capelli R, Minganti V, Bernhard M. 1987. Total mercury, organic mercury, copper, manganese, selenium, and zinc in Sarda sarda from the Guld of Genoa. Sci Total Environ 63:83-99.
Capelli R, Contardi V, Cosma B, Minganti V, Zanicchi G. 1983. A 4-year study on the distribution of some heavy metals in 5 marine organisms of the Ligurian Sea. Mar Chem 12(4):281-293.
Capelli R, Drava G, Siccardi C, De Pellegrini R, Minganti V. 2004. Study of the distribution of trace elements in six species of marine organisms of the Ligurian Sea (North-Western Mediterranean) - Comparison with previous findings. Ann Chim 94(7-8):533-546.
Cappon CJ. 1984. Content and chemical form of mercury and selenium in Lake Ontario salmon and trout. J Gt Lakes Res 10(4):429-434.
Carbonell G, Bravo JC, Fernandez C, Tarazona JV. 2009. A New Method for Total Mercury and Methyl Mercury Analysis in Muscle of Seawater Fish. Bull Environ Contam Toxicol 83(2):210-213.
Carvalho ML, Santiago S, Nunes ML. 2005. Assessment of the essential element and heavy metal content of edible fish muscle. Anal Bioanal Chem 382(2):426-432.
Chen CY, Chen MH. 2003. Investigation of Zn, Cu, Cd and Hg concentrations in the oyster of Chi-ku, Tai-shi and Tapeng Bay, southwestern Taiwan. J Food Drug Anal 11(1):32-38.
Chen MH, Chen CY, Chang SK, Huang SW. 2007. Total and organic mercury concentrations in the white muscles of swordfish (*Xiphias gladius*) from the Indian and Atlantic oceans. Food Addit Contam 24(9):969-975.
Cheung KC, Leung HM, Wong MH. 2008. Metal concentrations of common freshwater and marine fish from the Pearl River Delta, South China. Arch Environ Contam Toxicol 54(4):705-715.
Chou CL. 2007. A time series of mercury accumulation and improvement of dietary feed in net caged Atlantic salmon (*Salmo salar*). Mar Pollut Bull 54(6):720-725.
Chung SWC, Kwong KP, Tang ASP, Xiao Y, Ho PYY. 2008. Mercury and methylmercury levels in the main traded fish species in Hong Kong. Food Addit Contam Part B-Surveill 1(2):106-113.
Ciardullo S, Aureli F, Coni E, Guandalini E, Lost F, Raggi A, et al. 2008. Bioaccumulation potential of dietary arsenic, cadmium, lead, mercury, and selenium in organs and tissues of rainbow trout (*Oncorhyncus mykiss*) as a function of fish growth. J Agric Food Chem 56(7):2442-2451.
Cizdziel JV, Hinners TA, Heithmar EM. 2002. Determination of total mercury in fish tissues using combustion atomic absorption spectrometry with gold amalgamation. Water Air Soil Pollut 135(1-4):355-370.
Collings SE, Johnson MS, Leah RT. 1996. Metal contamination of angler-caught fish from the Mersey Estuary. Mar Environ Res 41(3):281-297.
Cross FA, Hardy LH, Jones NY, Barber RT. 1973. Relation between Total-Body Weight and Concentrations of Manganese, Iron, Copper, Zinc, and Mercury in White Muscle of Bluefish (*Pomatomus saltatrix*) and a Bathyl-Demersal Fish *Antimora rostrata*. Journal of the Fisheries Research Board of Canada 30(9):1287-1291.

Cutshall NH, Naidu JR, Pearcy WG. 1978. Mercury concentrations in pacific hake, *Merluccius productus (ayres)*, as a function of length and latitude. Science 200(4349):1489-1491.

Dabeka R, McKenzie AD, Forsyth DS, Conacher HBS. 2004. Survey of total mercury in some edible fish and shellfish species collected in Canada in 2002. Food Addit Contam 21(5):434-440.

Das YK, Aksoy A, Baskaya R, Duyar HA, Guvenc D, Boz V. 2009. Heavy Metal Levels of Some Marine Organisms Collected in Samsun and Sinop Coasts of Black Sea, in Turkey. Journal of Animal and Veterinary Advances 8(3):496-499.

Davies IM. 2012. Department of Agriculture and Fisheries for Scotland (DAFS), Fish and Shellfish Landed at Scottish Ports (1975-1976). Aberdeen, Scotland.

De Marco SG, Botte SE, Marcovecchio JE. 2006. Mercury distribution in abiotic and biological compartments within several estuarine systems from Argentina: 1980-2005 period. Chemosphere 65(2):213-223.

Del Gobbo LC, Archbold JA, Vanderlinden LD, Eckley CS, Diamond ML, Robson M. 2010. Risks and Benefits of Fish Consumption For Childbearing Women. Can J Diet Pract Res 71(1):41-45.

Delaware River Basin Commission. 2011. Fish and Shellfish Tissue Data, DRBC EPA Coastal 2000. Available: http://www.state.nj.us/drbc/fishtiss.htm [accessed 3 February 2011].

Della Torre C, Petochi T, Corsi I, Dinardo MM, Baroni D, Alcaro L, et al. 2010. DNA damage, severe organ lesions and high muscle levels of As and Hg in two benthic fish species from a chemical warfare agent dumping site in the Mediterranean Sea. Sci Total Environ 408(9):2136-2145.

Dellinger JA, Meyers RM, Gebhardt KJ, Hansen LK. 1996. The Ojibwa Health Study: Fish residue comparisons for Lakes Superior, Michigan, and Huron. Toxicol Ind Health 12(3-4):393-402.

Deshpande A, Bhendigeri S, Shirsekar T, Dhaware D, Khandekar RN. 2009. Analysis of heavy metals in marine fish from Mumbai Docks. Environ Monit Assess 159(1-4):493-500.

Deshpande AD, Draxler AFJ, Zdanowicz VS, Schrock ME, Paulson AJ. 2000. Contaminant Levels in Muscle of Four Species of Recreational Fish from the New York Bight Apex. NOAA Technical Memorandum NMFS-NE-157. NMFS-NE-157. Available: http://www.nefsc.noaa.gov/publications/tm/tm157/tm157.pdf [accessed 24 February 2009].

Dewailly E, Ayotte P, Lucas M, Blanchet C. 2007. Risk and benefits from consuming salmon and trout: A Canadian perspective. Food Chem Toxicol 45(8):1343-1348.

Dewailly E, Rouja P, Dallaire R, Pereg D, Tucker T, Ward J, et al. 2008. Balancing the risks and the benefits of local fish consumption in Bermuda. Food Addit Contam Part A-Chem 25(11):1328-1338.

Domingo JL, Bocio A, Falco G, Llobet JM. 2007. Benefits and risks of fish consumption Part I. A quantitative analysis of the intake of omega-3 fatty acids and chemical contaminants. Toxicology 230(2-3):219-226.

Doyon JF, Schetagne R, Verdon R. 1998. Different mercury bioaccumulation rates between sympatric populations of dwarf and normal lake whitefish (*Coregonus clupeaformis*) in the La Grande complex watershed, James Bay, Quebec. Biogeochemistry 40(2-3):203-216.

Duarte FA, Bizzi CA, Antes FG, Dressler VL, Flores EMD. 2009. Organic, inorganic and total mercury determination in fish by chemical vapor generation with collection on a gold gauze and electrothermal atomic absorption spectrometry. Spectroc Acta Pt B-Atom Spectr 64(6):513-519.

Easton MDL, Luszniak D, Von der Geest E. 2002. Preliminary examination of contaminant loadings in farmed salmon, wild salmon and commercial salmon feed. Chemosphere 46(7):1053-1074.

Elhamri H, Idrissi L, Coquery M, Azemard S, El Abidi A, Benlemlih M, et al. 2007. Hair mercury levels in relation to fish consumption in a community of the Moroccan Mediterranean coast. Food Addit Contam 24(11):1236-1246.

Elston R, Cake EW, Humphrey K, Isphording WC, Rensel JE. 2005. Dioxin and heavy-metal contamination of shellfish and sediments in St. Louis Bay, Mississippi and adjacent marine waters. J Shellfish Res 24(1):227-241.

Endo T, Haraguchi K. 2010. High mercury levels in hair samples from residents of Taiji, a Japanese whaling town. Mar Pollut Bull 60(5):743-747.

Escobar-Sanchez O, Galvan-Magana F, Rosiles-Martinez R. 2010. Mercury and Selenium Bioaccumulation in the Smooth Hammerhead Shark, *Sphyrna zygaena* Linnaeus, from the Mexican Pacific Ocean. Bull Environ Contam Toxicol 84(4):488-491.

Ethier ALM, Scheuhammer AM, Bond DE. 2008. Correlates of mercury in fish from lakes near Clyde Forks, Ontario, Canada. Environ Pollut 154(1):89-97.

Evans DW, Crumley PH. 2005. Mercury in Florida Bay fish: Spatial distribution of elevated concentrations and possible linkages to Everglades restoration. Bull Mar Sci 77(3):321-345.

Fabris G, Turoczy NJ, Stagnitti F. 2006. Trace metal concentrations in edible tissue of snapper, flathead, lobster, and abalone from coastal waters of Victoria, Australia. Ecotox Environ Safe 63(2):286-292.

Fairey R, Taberski K, Lamerdin S, Johnson E, Clark RP, Downing JW, et al. 1997. Organochlorines and other environmental contaminants in muscle tissues of sportfish collected from San Francisco Bay. Mar Pollut Bull 34(12):1058-1071.

Falandysz J. 1990. Mercury content of squid *Loligo opalescens*. Food Chem 38(3):171-177.

Ferreira AG, Faria VV, de Carvalho CEV, Lessa RPT, da Silva FMS. 2004. Total mercury in the night shark, *Carcharhinus signatus* in the western equatorial Atlantic Ocean. Braz Arch Biol Technol 47(4):629-634.

Fisher WS, Oliver LM, Winstead JT, Long ER. 2000. A survey of oysters *Crassostrea virginica* from Tampa Bay, Florida: associations of internal defense measurements with contaminant burdens. Aquat Toxicol 51(1):115-138.

Food Standards Agency. 2003. Methylmercury in imported Fish and Shellfish and Their Products, UK Farmed Fish and Their Products. London. Available: http://www.food.gov.uk/multimedia/pdfs/fsis40_2003.pdf [accessed 14 October 2003].

Food Standards Agency. 2006. Survey of Metals and Other Elements in Processed Fish and Shellfish. London. Available: http://www.food.gov.uk/multimedia/pdfs/fsis0806.pdf [accessed May 3 2008].

Forsyth DS, Casey V, Dabeka RW, McKenzie A. 2004. Methylmercury levels in predatory fish species marketed in Canada. Food Addit Contam 21(9):849-856.

Freeman HC, Horne DA. 1973. Sampling edible muscle of swordfish (*Xiphias gladius*) for total mercury analysis. Journal of the Fisheries Research Board of Canada 30(8):1251-1252.

Freeman HC, Shum G, Uthe JF. 1978. Selenium content in swordfish (*Xiphias gladius*) in relation to total mercury content. J Environ Sci Health Part A-Environ Sci Eng Toxic Hazard Subst Control 13(3):235-240.

Garcia-Hernandez J, Cadena-Cardenas L, Betancourt-Lozano M, Garcia-De-La-Parra LM, Garcia-Rico L, Marquez-Farias F. 2007. Total Mercury Content Found in Edible Tissues of Top Predator Fish From the Gulf of California, Mexico. Toxicological and Environmental Chemistry 89(3):507-522.

Garcia E, Carignan R. 2000. Mercury concentrations in northern pike (*Esox lucius*) from boreal lakes with logged, burned, or undisturbed catchments. Can J Fish Aquat Sci 57:129-135.

Gawlik B, Druges M, Bianchi M, Bortoli A, Kettrup A, Muntau H. 1997. TUNA FISH (T-30) - A new proficiency testing material for the determination of As and Hg in seafood. Fresenius J Anal Chem 358(3):441-445.

Gerhart EH. 1977. Concentrations of total mercury in several fishes from Delaware Bay, 1975. Pesticides Monitoring Journal 11(3):132-133.

Gerstenberger SL, Martinson A, Kramer JL. 2010. An evaluation of mercury concentrations in three brands of canned tuna. Environ Toxicol Chem 29(2):237-242.

Gilmartin M, Revelante N. 1975. Concentration of Mercury, Copper, Nickel, Silver, Cadmium, and Lead in Northern Adriatic Anchovy, *Engraulis encrasicholus*, and Sardine, *Sardina pilchardus*. Fishery Bulletin 73(1):193-201.

Green-Ruiz C, Ruelas-Inzunza J, Paez-Osuna F. 2005. Mercury in surface sediments and benthic organisms from Guaymas Bay, east coast of the Gulf of California. Environ Geochem Health 27(4):321-329.

Green NW, Knutzen J. 2003. Organohalogens and metals in marine fish and mussels and some relationships to biological variables at reference localities in Norway. Mar Pollut Bull 46(3):362-374.

Greenfield BK, Jahn A. 2010. Mercury in San Francisco Bay forage fish. Environ Pollut 158(8):2716-2724.

Greig RA, Wenzloff DR, Mackenzie CL, Merrill AS, Zdanowicz VS. 1978. Trace metals in sea scallops, *Placopecten magellanicus*, from eastern United States. Bull Environ Contam Toxicol 19(3):326-334.

Gunsen U. 2004. The residue levels of some toxic metals in different fish species. Indian Veterinary Journal 81(12):1339-1341.

Gutierrez AJ, Lozano G, Gonzalez T, Reguera JI, Hardisson A. 2006. Mercury content in tinned molluscs (mussel, cockle, variegated scallop, and razor shell) normally consumed in Spain, 2005. J Food Prot 69(9):2237-2240.

Haines TA, Komov V, Jagoe CH. 1992. Lake acidity and mercury content of fish in Darwin National Reserve, Russia. Environ Pollut 78(1-3):107-112.

Hajeb P, Jinap S, Fatimah AB, Jamilah B. 2010. Methylmercury in marine fish from Malaysian waters and its relationship to total mercury content. Int J Environ Anal Chem 90(10):812-820.

Hajeb P, Jinap S, Ismail A, Fatimah AB, Jamilah B, Rahim MA. 2009. Assessment of mercury level in commonly consumed marine fishes in Malaysia. Food Control 20(1):79-84.

Hall AS, Teeny FM, Lewis LG, Hardman WH, Gauglitz EJ. 1976. Mercury in fish and shellfish of northeast pacific. 1. Pacific Halibut, *Hippoglossus stenolepis*. Fishery Bulletin 74(4):783-789.

Hall RA, Zook EG, Meaburn GM. 1978. National Marine Fisheries Service Survey of Trace Elements in the Fishery Resources. NOAA Technical Report NMFS SSRF-721. TR 721. Rockville, MD:National Oceanic and Atmospheric Administration, National Marine Fisheries Service.

Hammerschmidt CR, Fitzgerald WF. 2006. Bioaccumulation and trophic transfer of methylmercury in Long Island Sound. Arch Environ Contam Toxicol 51(3):416-424.

Harding G, Dalziel J, Vass P. 2005. Prevalence and bioaccumulation of methylmercury in the food web of the Bay of Fundy, Gulf of Maine. In: The Changing Bay of Fundy - Beyond 400 Years Proceedings of the 6th Bay of Fundy Workshop (Percy JA, Evans AJ, Wells PG, Rolston SJ, eds). Cornwalis, Nova Scotia. September 29 - October 2, 2004. Environment Canada, Atlantic Region, 76-77.

Hardisson A, Padron AG, de Bonis A, Sierra A. 1999. Determination of mercury in fish by cold vapor atomic absorption spectrometry. Atom Spectrosc 20(5):191-193.

Health Canada. 2007. Human Health Risk Assessment of Mercury in Fish and Health Benefits of Fish Consumption. Ottawa, Ontario. Available: http://www.hc-sc.gc.ca/fn-an/pubs/mercur/merc_fish_poisson-eng.php [accessed 31 October 2011].

Hellou J, Fancey LL, Payne JF. 1992a. Concentrations of 24 elements in bluefin tuna, *Thunnus thynnus* from the northwest Atlantic. Chemosphere 24(2):211-218.

Hellou J, Warren WG, Payne JF, Belkhode S, Lobel P. 1992b. Heavy metals and other elements in 3 tissues of cod, *Gadus morhua* from the northwest. Atlantic Mar Pollut Bull 24(9):452-458.

Herreros MA, Inigo-Nunez S, Sanchez-Perez E, Encinas T, Gonzalez-Bulnes A. 2008. Contribution of fish consumption to heavy metals exposure in women of childbearing age from a Mediterranean country (Spain). Food Chem Toxicol 46(5):1591-1595.

Horwitz R, Ashley J, Overbeck P, Velinsky D. 2005. Final Report: Routine Monitoring Program for Toxics in Fish. Trenton, NJ. Available: http://www.state.nj.us/dep/dsr/final-report-routinemonitoing5-05.pdf [accessed 24 October 2007].

Hueter RE, Fong WG, Henderson G, French MF, Manire CA. 1995. Methylmercury concentration in shark muscle by species, size and distribution of sharks in Florida coastal waters. Water Air Soil Pollut 80(1-4):893-899.

Ikem A, Egiebor NO. 2005. Assessment of trace elements in canned fishes (mackerel, tuna, salmon, sardines and herrings) marketed in Georgia and Alabama (United States of America). Journal of Food Composition and Analysis 18(8):771-787.

International Pacific Halibut Commission. 2003. Methylmercury and Heavy Metal Contaminant Levels in Alaskan Halibut. Available: http://www.iphc.washington.edu/halcom/pubs/rara/2003rara/2k308RARA.pdf [accessed 19 July 2004].

Jackson TA. 1991. Biological and environmental control of mercury accumulation by fish in lakes and reservoirs of Northern Manitoba, Canada. Can J Fish Aquat Sci 48(12):2449-2470.

Jackson TA, Whittle DM, Evans MS, Muir DCG. 2008. Evidence for mass-independent and mass-dependent fractionation of the stable isotopes of mercury by natural processes in aquatic ecosystems. Appl Geochem 23(3):547-571.

Jaeger I, Hop H, Gabrielsen GW. 2009. Biomagnification of mercury in selected species from an Arctic marine food web in Svalbard. Sci Total Environ 407(16):4744-4751.

Japanese Ministry of Health. 2003. Results of Mercury/Methylmercury in Fishes (Provisional Translation). Available: http://www.mhlw.go.jp/english/wp/other/councils/mercury/index.html [accessed 28 January 2011].

Jardine LB, Burt MDB, Arp PA, Diamond AW. 2009. Mercury comparisons between farmed and wild Atlantic salmon (*Salmo salar* L.) and Atlantic cod (*Gadus morhua* L.). Aquac Res 40(10):1148-1159.

Jasmine GI, Rajagopalsamy CBT, Jeyachandran P. 1989. Total mercury content of Indian Squid *Loligo duvauceli orbigny* from Tuticorin waters, south east coast of India. Indian J Mar Sci 18(3):219-220.

Jewett SC, Naidu AS. 2000. Assessment of heavy metals in red king crabs following offshore placer gold mining. Mar Pollut Bull 40(6):478-490.

Jokai Z, Abranko L, Fodor P. 2005. SPME-GC-pyrolysis-AFS determination of methylmercury in marine fish products by alkaline sample preparation and aqueous phase phenylation derivatization. J Agric Food Chem 53(14):5499-5505.

Julshamn K, Brenna J. 2002. Determination of mercury in seafood by flow injection-cold vapor atomic absorption spectrometry after microwave digestion: NMKL Interlaboratory Study. J AOAC Int 85(3):626-631.

Julshamn K, Grosvik BE, Nedreaas K, Maage A. 2006. Mercury concentration in fillets of Greenland halibut (*Reinhardtius hippoglossoides*) caught in the Barents Sea in January 2006. Sci Total Environ 372(1):345-349.

Julshamn K, Lundebye AK, Heggstad K, Berntssen MHG, Boe B. 2004. Norwegian monitoring programme on the inorganic and organic contaminants in fish caught in the Barents Sea, Norwegian Sea and North Sea, 1994-2001. Food Addit Contam 21(4):365-376.

Juresa D, Blanusa M. 2003. Mercury, arsenic, lead and cadmium in fish and shellfish from the Adriatic Sea. Food Addit Contam 20(3):241-246.

Kai N, Ueda T, Takeda Y, Kataoka A. 1987. Accumulation of mercury and selenium in blue marlin. Nippon Suisan Gakkaishi 53(9):1697-1697.

Kamps LR, Carr R, Miller H. 1972. Total mercury - monomethylmercury content of several species of fish. Bull Environ Contam Toxicol 8(5):273-279.

Kaneko JJ, Ralston NVC. 2007. Selenium and mercury in pelagic fish in the central north pacific near Hawaii. Biol Trace Elem Res 119:242-254.

Kannan K, Smith RG, Lee RF, Windom HL, Heitmuller PT, Macauley JM, et al. 1998. Distribution of total mercury and methyl mercury in water, sediment, and fish from south Florida estuaries. Arch Environ Contam Toxicol 34(2):109-118.

Karouna-Renier NK, Snyder RA, Allison JG, Wagner MG, Rao KR. 2007. Accumulation of organic and inorganic contaminants in shellfish collected in estuarine waters near Pensacola, Florida: Contamination profiles and risks to human consumers. Environ Pollut 145(2):474-488.

Kawaguchi T, Porter D, Bushek D, Jones B. 1999. Mercury in the American oyster *Crassostrea virginica* in South Carolina, USA, and public health concerns. Mar Pollut Bull 38(4):324-327.

Kehrig HDA, Costa M, Moreira I, Malm O. 2001. Methylmercury and total mercury in estuarine organisms from Rio de Janeiro, Brazil. Environ Sci Pollut Res 8(4):275-279.

Kelso JRM, Frank R. 1974. Organochlorine residues, mercury, copper and cadmium in yellow perch, white bass and smallmouth bass, Long Point Bay, Lake Erie. Trans Am Fish Soc 103(3):577-581.

Khansari FE, Ghazi-Khansari M, Abdollahi M. 2005. Heavy metals content of canned tuna fish. Food Chem 93(2):293-296.

Kidd KA, Hesslein RH, Fudge RJP, Hallard KA. 1995. The influence of trophic level as measured by delta N-15 on mercury concentrations in freshwater organisms. Water Air Soil Pollut 80(1-4):1011-1015.

Knight HT, Olson LJ. 1974. Mercury distribution in american smelt from Lake Michigan. Am Midl Nat 91(2):451-452.

Knobeloch LM, Ziarnik M, Anderson HA, Dodson VN. 1995. Imported sea bass as a source of mercury exposure - a Wisconsin case study. Environ Health Perspect 103(6):604-606.

Knowles TG, Farrington D, Kestin SC. 2003. Mercury in UK imported fish and shellfish and UK-farmed fish and their products. Food Addit Contam 20(9):813-818.

Kojadinovic J, Potier M, Le Corre M, Cosson RP, Bustamante P. 2006. Mercury content in commercial pelagic fish and its risk assessment in the Western Indian Ocean. Sci Total Environ 366(2-3):688-700.

Kojadinovic J, Potier M, Le Corre M, Cosson RP, Bustamante P. 2007. Bioaccumulation of trace elements in pelagic fish from the Western Indian Ocean. Environ Pollut 146(2):548-566.

Koli AK, Williams WR, McClary EB, Wright EL, Burrell TM. 1977. Mercury Levels in Freshwater Fish of State of South-Carolina. Bull Environ Contam Toxicol 17(1):82-89.

Kraepiel AML, Keller K, Chin HB, Malcolm EG, Morel FMM. 2003. Sources and variations of mercury in tuna. Environ Sci Technol 37(24):5551-5558.

Krystek P, Ritsema R. 2004. Determination of methylmercury and inorganic mercury in shark fillets. Appl Organomet Chem 18(12):640-645.

Kumar M, Aalbersberg B, Mosley L. 2004. IAS Technical Report Number: 2004/03, Mercury Levels in Fijian Seafoods and Potential Health Implications, Report for World Health Organization.

Kutter VT, Mirlean N, Baisch PRM, Kutter MT, Silva E. 2009. Mercury in freshwater, estuarine, and marine fishes from Southern Brazil and its ecological implication. Environ Monit Assess 159(1-4):35-42.

Kwoczek M, Szefer P, Hac E. 2006. Essential and toxic elements in seafood available in Poland from different geographical regions. J Agric Food Chem 54(8):3015-3024.

Laperdina TG, Askarova OB, Papina TS, Eirikh SS, Sorokovikova LM. 1997. Methodological features of the determination of mercury in fish samples (Using fish from the Kureiskoe Reservoir as an example). J Anal Chem 52(6):584-589.

Legrand M, Arp P, Ritchie C, Chan HM. 2005. Mercury exposure in two coastal communities of the Bay of Fundy, Canada. Environ Res 98(1):14-21.

Levine KE, Levine MA, Weber FX, Henderson JP, Grohse PM. 2005. Mercury in an assortment of processed and unprocessed seafood samples. Bull Environ Contam Toxicol 74(5):973-979.

Lewis MA, Quarles RL, Dantin DD, Moore JC. 2004. Evaluation of a Florida coastal golf complex as a local and watershed source of bioavailable contaminants. Mar Pollut Bull 48(3-4):254-262.

Licata P, Trombetta D, Cristani M, Naccari C, Martino D, Calo M, et al. 2005. Heavy metals in liver and muscle of bluefin tuna (*Thunnus thynnus*) caught in the straits of Messina (Sicily, Italy). Environ Monit Assess 107(1-3):239-248.

Linko RR, Terho K. 1977. Occurrence of methyl mercury in pike and baltic herring from Turku Archipelago. Environ Pollut 14(3):227-235.

Locascio JV, Rudershausen PJ. 2000. An Evaluation of Mercury Levels in Spotted Seatrout in Torpon Bay, J.N. "Ding" Darling Wildlife Rufuge, Sanibel, Florida, With Reference to Previous Studies. Biological Sciences 63(4):256-260.

Lockhart WL, Stern GA, Low G, Hendzel M, Boila G, Roach P, et al. 2005. A history of total mercury in edible muscle of fish from lakes in northern Canada. Sci Total Environ 351:427-463.

Lourenco HM, Anacleto P, Afonso C, Ferraria V, Martins MF, Carvalho ML, et al. 2009. Elemental composition of cephalopods from Portuguese continental waters. Food Chem 113(4):1146-1153.

Lowenstein J, Burger J, Jeitner C, Amato G, Kolokotronis S, Gochfeld M. 2010. DNA Barcodes Reveal Species-specific Mercury Levels in Tuna Sushi That Pose a Health Risk to Consumers. Biology Letters 6(5):692-695.

Lower Duwamish Waterway Group. 2005. Lower Duwamish Waterway Cleanup: Fish and Crab Tissue Data Report. Available: http://www.ldwg.org/assets/fish_crab_tissue/appendix_a-data_tables_final.pdf [accessed 2 December 2010].

Luckhurst BE, Prince ED, Llopiz JK, Snodgrass D, Brothers EB. 2006. Evidence of blue marlin (*Makaira nigricans*) spawning in Bermuda waters and elevated mercury levels in large specimens. Bull Mar Sci 79(3):691-704.

Madany IM, Wahab AAA, AlAlawi Z. 1996. Trace metals concentrations in marine organisms from the coastal areas of Bahrain, Arabian Gulf. Water Air Soil Pollut 91(3-4):233-248.

Madenjian C, O'Connor D. 2008. Trophic Transfer Efficiency of Mercury to Lake Whitefish *Coregonus clupeaformis* from its Prey. Bull Environ Contam Toxicol 81(6):566-570.

Magalhaes MC, Costa V, Menezes GM, Pinho MR, Santos RS, Monteiro LR. 2007. Intra- and inter-specific variability in total and methylmercury bioaccumulation by eight marine fish species from the Azores. Mar Pollut Bull 54(10):1654-1662.

Marcovecchio JE, Moreno VJ, Perez A. 1986. Bio-magnification of total mercury in Bahia Blanca Estuary shark. Mar Pollut Bull 17(6):276-278.

Marcovecchio JE, Moreno VJ, Perez A. 1991. Metal accumulation in tissues of sharks from the Bahia Blanca Estuary, Argentina. Mar Environ Res 31(4):263-274.

Marsico ET, Machado MES, Knoff M, Clemente SCS. 2007. Total mercury in sharks along the southern Brazilian Coast. Arq Bras Med Vet Zootec 59(6):1593-1596.

Mason RP, Heyes D, Sveinsdottir A. 2006. Methylmercury concentrations in fish from tidal waters of the Chesapeake Bay. Arch Environ Contam Toxicol 51(3):425-437.

McArthur T, Butler ECV, Jackson GD. 2003. Mercury in the marine food chain in the Southern Ocean at Macquarie Island: an analysis of a top predator, Patagonian toothfish (*Dissostichus eleginoides*) and a mid-trophic species, the warty squid (*Moroteuthis ingens*). Polar Biol 27(1):1-5.

McKelvey W, Chang M, Arnason J, Jeffery N, Kricheff J, Kass D. 2010. Mercury and polychlorinated biphenyls in Asian market fish: A response to results from mercury biomonitoring in New York City. Environ Res 110(7):650-657.

Meador JP, Ernest DW, Kagley AN. 2005. A comparison of the non-essential elements cadmium, mercury, and lead found in fish and sediment from Alaska and California. Sci Total Environ 339(1-3):189-205.

Menasveta P, Siriyong R. 1977. Mercury content of severeal predacious fish in Andaman Sea. Mar Pollut Bull 8(9):200-204.

Mendez E, Giudice H, Pereira A, Inocente G, Medina D. 2001. Total mercury content - Fish weight relationship in swordfish (*Xiphias gladius*) caught in the southwest Atlantic Ocean. Journal of Food Composition and Analysis 14(5):453-460.

Miller GE, Rowland FS, Steinkru.Fj, Grant PM, Guinn VP, Kishore R. 1972. Mercury concentration in museum specimens of tuna and swordfish. Science 175(4026):1121-1122.

Miller TJ, Jude DJ. 1984. Organochlorine pesticides, PBBs, and mercury in round whitefish fillets from Saginaw Bay, Lake Huron, 1977-1978. J Gt Lakes Res 10(2):215-220.

Ministry of Agriculture Fisheries and Food. 1998. Concentrations of Metals and Other Elements in Marine fish and Shellfish. Available: http://archive.food.gov.uk/maff/archive/food/infsheet/1998/no151/151fish.htm [accessed 18 March 2011].

Mol JH, Ramlal JS, Lietar C, Verloo M. 2001. Mercury contamination in freshwater, estuarine, and marine fishes in relation to small-scale gold mining in Suriname, South America. Environ Res 86(2):183-197.

Monteiro LR, Lopes HD. 1990. Merucy content of swordfish, *Xiphias gladius*, in relation to length, weight, age, and sex. Mar Pollut Bull 21(6):293-296.

Mueller CS, Ramelow GJ, Beck JN. 1989. Mercury in the Calcasieu River Lake Complex, Louisiana. Bull Environ Contam Toxicol 42(1):71-80.

Nadal M, Ferre-Huguet N, Marti-Cid R, Schuhmacher M, Domingo JL. 2008. Exposure to metals through the consumption of fish and seafood by the population living near the Ebro River in Catalonia, Spain: Health risks. Hum Ecol Risk Assess 14(4):780-795.

Nakagawa R, Yumita Y, Hiromoto M. 1997. Total mercury intake from fish and shellfish by Japanese people. Chemosphere 35(12):2909-2913.

Nakao M, Seoka M, Tsukamasa Y, Kawasaki K, Ando M. 2007. Possibility for decreasing of mercury content in bluefin tuna *Thunnus orientalis* by fish culture. Fisheries Science 73(3):724-731.

Nakao M, Seoka M, Nakatani M, Okada T, Miyashita S, Tsukamasa Y, et al. 2009. Reduction of mercury levels in cultured bluefin tuna, *Thunnus orientalis*, using feed with relatively low mercury levels. Aquaculture 288(3-4):226-232.

National Marine Fisheries Service. 1975. Southwest Fisheries Center Administrative Report No. 2H, 1975, Mercury in the Pacific Blue Marlin.

Nfon E, Cousins IT, Jarvinen O, Mukherjee AB, Verta M, Broman D. 2009. Trophodynamics of mercury and other trace elements in a pelagic food chain from the Baltic Sea. Sci Total Environ 407(24):6267-6274.

NOAA (National Oceanic and Atmospheric Administration). 2008. National Status and Trends Mussel Watch Program. Available: http://nsandt.noaa.gov/ [accessed 9 September 2008].

Oh KS, Suh J, Park S, Paek OA, Yoon HJ, Kim HY, et al. 2008. Mercury and methylmercury levels in marine fish species from Korean retail markets. Food Sci Biotechnol 17(4):819-823.

Orban E, Nevigato T, Di Lena G, Masci M, Casini I, Garnbelli L, et al. 2008. New trends in the seafood market. Sutchi catfish (*Pangasius hypophthalmus*) fillets from Vietnam: Nutritional quality and safety aspects. Food Chem 110(2):383-389.

Ozden O. 2010. Seasonal differences in the trace metal and macrominerals in shrimp (*Parapenaus longirostris*) from Marmara Sea. Environ Monit Assess 162(1-4):191-199.

Padula DJ, Daughtry BJ, Nowak BF. 2008. Dioxins, PCBs, metals, metalloids, pesticides and antimicrobial residues in wild and farmed Australian southern bluefin tuna (*Thunnus maccoyii*). Chemosphere 72(1):34-44.

Panutrakul S, Khamdech S, Kerdthong P, Senanan W, Tangkrock-Olan N, Alcivar-Warren A. 2007. Heavy metals in wild banana prawn (*Fenneropenaeus merguiensis* de Man, 1888) from Chantaburi and Trat provinces, Thailand. J Shellfish Res 26(4):1193-1202.

Papetti P, Rossi G. 2009. Heavy metals in the fishery products of low Lazio and the use of metallothionein as a biomarker of contamination. Environ Monit Assess 159(1-4):589-598.

Park J, Presley BJ. 1997. Trace metals contamination of sediments and organisms from the Swan Lake area of Galveston Bay. Environ Pollut 98(2):209-221.

Pastor A, Hernandez F, Peris MA, Beltran J, Sancho JV, Castillo MT. 1994. Levels of heavy metals in some marine organisms from the western Mediterranean area (Spain). Mar Pollut Bull 28(1):50-53.

Paul MC, Toia RF, von Nagy-Felsobuki EI. 2003. A novel method for the determination of mercury and selenium in shark tissue using high-resolution inductively coupled plasma-mass spectrometry. Spectroc Acta Pt B-Atom Spectr 58(9):1687-1697.

Payne EJ, Taylor DL. 2010. Effects of Diet Composition and Trophic Structure on Mercury Bioaccumulation in Temperate Flatfishes. Arch Environ Contam Toxicol 58(2):431-443.
Penedo de Pinho A, Davee Guimaraes JR, Martins AS, Costa PAS, Olavo G, Valentin J. 2002. Total mercury in muscle tissue of five shark species from Brazilian offshore waters: effects of feeding habit, sex, and length. Environ Res 89(3):250-258.
Perello G, Marti-Cid R, Llobet JM, Domingo JL. 2008. Effects of Various Cooking Processes on the Concentrations of Arsenic, Cadmium, Mercury, and Lead in Foods. J Agric Food Chem 56(23):11262-11269.
Petersen A, Mortensen GK. 1994. Trace elements in shellfish on the Danish market. Food Addit Contam 11(3):365-373.
Piraino MN, Taylor DL. 2009. Bioaccumulation and trophic transfer of mercury in striped bass (*Morone saxatilis*) and tautog (*Tautoga onitis*) from the Narragansett Bay (Rhode Island, USA). Mar Environ Res 67(3):117-128.
Plessi M, Bertelli D, Monzani A. 2001. Mercury and selenium content in selected seafood. Journal of Food Composition and Analysis 14(5):461-467.
Polak-Juszczak L. 2009. Temporal trends in the bioaccumulation of trace metals in herring, sprat, and cod from the southern Baltic Sea in the 1994-2003 period. Chemosphere 76(10):1334-1339.
Poperechna N, Heumann KG. 2005. Simultaneous multi-species determination of trimethyllead, monomethylmercury and three butyltin compounds by species-specific isotope dilution GC-ICP-MS in biological samples. Anal Bioanal Chem 383(2):153-159.
Rahman SA, Wood AK, Sarmani S, Majid AA. 1997. Determination of mercury and organic mercury contents in Malaysian seafood. J Radioanal Nucl Chem 217(1):53-56.
Ramlal PS, Bugenyi FWB, Kling GW, Nriagu JO, Rudd JWM, Campbell LM. 2003. Mercury concentrations in water, sediment, and biota from Lake Victoria, East Africa. J Gt Lakes Res 29:283-291.
Rasmussen RS, Morrissey MT. 2007. Effects of canning on total mercury, protein, lipid, and moisture content in troll-caught albacore tuna (*Thunnus alalunga*). Food Chem 101(3):1130-1135.
Ray S, Jessop BM, Coffin J, Swetnam DA. 1984. Mercury and Polychlorinated-Biphenyls in Striped Bass (*Morone saxatilis*) from 2 Nova-Scotia Rivers. Water Air Soil Pollut 21(1-4):15-23.
Raymond B, Rossmann R. 2009. Total and methyl mercury accumulation in 1994-1995 Lake Michigan lake trout and forage fish. J Gt Lakes Res 35(3):438-446.
Rider S, Adams D. 2000. Mercury Concentrations in Spotted Seatrout from Northwest Florida. Gulf of Mexico Science 2:97-103.
Riget F, Moller P, Dietz R, Nielsen TG, Asmund G, Strand J, et al. 2007. Transfer of mercury in the marine food web of West Greenland. J Environ Monit 9(8):877-883.
Rivers JB, Pearson JE, Shultz CD. 1972. Total and organic mercury in marine fish. Bull Environ Contam Toxicol 8(5):257-265.
Rolfhus KR, Sandheinrich MB, Wiener JG, Bailey SW, Thoreson KA, Hammerschmidt CR. 2008. Analysis of fin clips as a nonlethal method for monitoring mercury in fish. Environ Sci Technol 42(3):871-877.
Romeo M, Siau Y, Sidoumou Z, Gnassia-Barelli M. 1999. Heavy metal distribution in different fish species from the Mauritania coast. Sci Total Environ 232(3):169-175.
Ruelas-Inzunza J, Paez-Osuna F. 2005. Mercury in fish and shark tissues from two coastal lagoons in the gulf of California, Mexico. Bull Environ Contam Toxicol 74(2):294-300.
Ruelas-Inzunza J, Garcia-Rosales SB, Paez-Osuna F. 2004. Distribution of mercury in adult penaeid shrimps from Altata-Ensenada del Pabellon lagoon (SE Gulf of California). Chemosphere 57(11):1657-1661.
Ruelas-Inzunza J, Meza-Lopez G, Paez-Osuna F. 2008. Mercury in fish that are of dietary importance from the coasts of Sinaloa (SE Gulf of California). Journal of Food Composition and Analysis 21(3):211-218.

Sahuquillo I, Lagarda MJ, Silvestre MD, Farre R. 2007. Methylmercury determination in fish and seafood products and estimated daily intake for the Spanish population. Food Addit Contam 24(8):869-876.

Sajwan KS, Kumar KS, Paramasivam S, Compton SS, Richardson JP. 2008. Elemental status in sediment and American oyster collected from Savannah marsh/estuarine ecosystem: A preliminary assessment. Arch Environ Contam Toxicol 54(2):245-258.

San Francisco Estuary Institute. 2007. California Bay – Delta Authority Fish Mercury Project: Year 2 Annual Report (Sport Fish Sampling and Analysis). Contribution no. 535. Available: http://legacy.sfei.org/rmp/data/rmpfishtissue.htm [accessed 10 April 2008].

San Francisco Public Utilities Commission. 2006. Southwest Ocean Outfall Regional Monitoring Program, Eight Year Summary Report, 1997-2004 San Francisco. Available: http://sfwater.org/main.cfm/MC_ID/4/MSC_ID/83 [accessed 27 January 2011].

Santerre CR, Bush PB, Xu DH, Lewis GW, Davis JT, Grodner RM, et al. 2001. Metal residues in farm-raised channel catfish, rainbow trout, and red swamp crayfish from the southern US. J Food Sci 66(2):270-273.

Santoyo MM, Figueroa JAL, Wrobel K. 2009. Analytical speciation of mercury in fish tissues by reversed phase liquid chromatography-inductively coupled plasma mass spectrometry with Bi3+ as internal standard. Talanta 79(3):706-711.

Schetagne R, Doyon JF, Fournier JJ. 2000. Export of mercury downstream from reservoirs. Sci Total Environ 260(1-3):135-145.

Scheuhammer AM, Graham JE. 1999. The bioaccumulation of mercury in aquatic organisms from two similar lakes with differing pH. Ecotoxicology 8(1):49-56.

Schuler LJ, Howell JP, Heagler MG. 2000. Mercury concentrations in Louisiana and Chinese crayfish. Bull Environ Contam Toxicol 64(1):27-32.

Senn DB, Chesney EJ, Blum JD, Bank MS, Maage A, Shine JP. 2010. Stable Isotope (N, C, Hg) Study of Methylmercury Sources and Trophic Transfer in the Northern Gulf of Mexico. Environ Sci Technol 44(5):1630-1637.

Shim SM, Dorworth LE, Lasrado JA, Santerre CR. 2004. Mercury and fatty acids in canned tuna, salmon, and mackerel. J Food Sci 69(9):C681-C684.

Shim SM, Lasrado JA, Dorworth LE, Santerre CR. 2005. Mercury and Omega-3 fatty acids in retail fish sandwiches. J Food Prot 68(3):633-635.

Shomura R, Craig W. 1974. Mercury in Several Species of Billfishes Taken Off Hawaii and Southern California Kailua-Kona, Hawaii:Proceedings of the International Billfish Symposium.

Shultz CD, Crear D. 1976. Distribution of total and organic mercury in 7 tissues of Pacific blue marlin, *Makaira nigricans*. Pacific Science 30(2):101-107.

Shultz CD, Ito BM. 1979. Mercury and selenium in blue marlin, *Makaira nigricans*, from the Hawaiian Islands. Fishery Bulletin 76(4):872-879.

Soegianto A, Moehammadi N, Irawan B, Affandi M, Hamami. 2010. Mercury concentrations in edible species harvested from Gresik coast, Indonesia and its health risk assessment. Cah Biol Mar 51(1):1-8.

Soto-Jimenez MF, Amezcua F, Gonzalez-Ledesma R. 2010. Nonessential Metals in Striped Marlin and Indo-Pacific Sailfish in the Southeast Gulf of California, Mexico: Concentration and Assessment of Human Health Risk. Arch Environ Contam Toxicol 58(3):810-818.

State of Alaska Department of Environmental Conservation. 2009. Total Mercury Concentrations in Alaskan Fishes. Available: http://dec.alaska.gov/eh/docs/vet/metals%2009-11-19.pdf [accessed 26 January 2011].

State of Delaware. 2010. Department of Natural Resources and Environmental Control Open Files. Available: http://www.dnrec.state.de.us/fw/advisory.htm [accessed 29 November 2010].

State of Louisiana. 2011. Department of Environmental Quality Mercury Fish Tissue Data 2005-2010 (Data file). Available: http://www.deq.louisiana.gov/portal/default.aspx?tabid=1633 [accessed 4 February 2011].

State of Maryland. 2007. Toxics Data Sets. Available: http://archive.chesapeakebay.net/data/historicaldb/toxicsmain.htm [accessed 24 October 2007].

State of Michigan. 2011. Fish Contaminant Monitoring Program Online Database. Available: http://www.deq.state.mi.us/fcmp/ [accessed 25 February 2011].

State of New Jersey. 2004. Routine Monitoring For Toxics in Fish Program Available: http://www.state.nj.us/dep/dsr/task1.pdf (Task I- Appendix I. Draft Summary of Chemical Contaminant Concentrations) and http://www.state.nj.us/dep/dsr/task2.pdf (Task 2) [accessed 29 November 2011].

State of New Jersey. 2008. Routine Monitoring Program for Toxics in Fish: Year 3 Raritan River Region. Available: http://www.state.nj.us/dep/dsr/fishmonitoring-year3.pdf [accessed 29 November 2011].

State of North Carolina. 2011. Statewide Fish Tissue Metals Results Available: http://portal.ncdenr.org/web/wq/ess/bau/fish-tissue-data, for 1990-2010 [accessed 26 January 2011].

State of Virginia. 2009. Department of Environmental Quality Fish Tissue Results Summary. Available: http://www.deq.state.va.us/fishtissue/fishtissue.html [accessed 21 July 2009].

Storelli MM, Marcotrigiano GO. 2001. Total mercury levels in muscle tissue of swordfish (*Xiphias gladius*) and bluefin tuna (*Thunnus thynnus*) from the Mediterranean Sea (Italy). J Food Prot 64(7):1058-1061.

Storelli MM, Stuffler RG, Marcotrigiano GO. 1998. Total mercury in muscle of benthic and pelagic fish from the South Adriatic Sea (Italy). Food Addit Contam 15(8):876-883.

Storelli MM, Stuffler RG, Marcotrigiano GO. 2002. Total and methylmercury residues in tuna-fish from the Mediterranean sea. Food Addit Contam 19(8):715-720.

Storelli MM, Giacominelli-Stuffler R, Storelli A, Marcotrigiano GO. 2005. Accumulation of mercury, cadmium, lead and arsenic in swordfish and bluefin tuna from the Mediterranean Sea: A comparative study. Mar Pollut Bull 50(9):1004-1007.

Storelli MM, Giacominelli-Stuffler R, Storelli A, Marcotrigiano GO. 2006. Cadmium and mercury in cephalopod molluscs: Estimated weekly intake. Food Addit Contam 23(1):25-30.

Storelli MM, Barone G, Piscitelli G, Marcotrigiano GO. 2007. Mercury in fish: Concentration vs. fish size and estimates of mercury intake. Food Addit Contam 24(12):1353-1357.

Storelli MM, Garofalo R, Giungato D, Giacominelli-Stuffler R. 2010. Intake of essential and non-essential elements from consumption of octopus, cuttlefish and squid. Food Addit Contam Part B-Surveill 3(1):14-18.

Storelli MM, Giacominelli-Stuffler R, Storelli A, D'Addabbo R, Palermo C, Marcotrigiano GO. 2003. Survey of total mercury and methylmercury levels in edible fish from the Adriatic Sea. Food Addit Contam 20(12):1114-1119.

Strom DG, Graves GA. 2001. A comparison of mercury in estuarine fish between Florida Bay and the Indian River Lagoon, Florida, USA. Estuaries 24(4):597-609.

Suk SH, Smith SE, Ramon DA. 2009. Bioaccumulation of mercury in pelagic sharks from the northeast Pacific Ocean. CalCOFI Rep., Vol. 50, 2009. La Jolla, CA. Available: http://calcofi.org/publications/calcofireports/v50/172-177_Suk.pdf [accessed 3 May 2011].

Tahan JE, Sanchez JM, Granadillo VA, Cubillan HS, Romero RA. 1995. Concentrations of total Al, Cr, Cu, Fe, Hg, Na, Pb, and Zn in commercial canned seafood determined by atomic spectrometric means after mineralization by microwave heating. J Agric Food Chem 43(4):910-915.

Tam SYK, Mok CS. 1991. Metallic contamination in oyster and other seafood in Hong Kong. Food Addit Contam 8(3):333-342.

Teeny FM, Hall AS, Gauglitz EJ. 1974. Reduction of mercury in sablefish (*Anoplopoma fimbria*) and use of treated flesh in smoked products. Marine Fisheries Review 36(5):15-17.

Thieleke J. 1973. Mercury Levels in five Species of Commercially Important Pelagic fish Taken From the Pacific Ocean Near Hawaii [PhD Dissertation]. Madison, WI: University of Wisconsin, Madison.

Thomson B, Lee L. 2009. Mercury content in imported fin fish. Available: http://www.foodsafety.govt.nz/elibrary/industry/Mercury_Content-Quantifies_Residues.pdf [accessed 2 December 2010].

Torres-Escribano S, Velez D, Montoro R. 2010. Mercury and methylmercury bioaccessibility in swordfish. Food Addit Contam Part A-Chem 27(3):327-337.

Tyrell L, McHugh B, Glynn D, Twomey M, Joyce E, Costello J, et al. 2005. Trace Metal Concentrations in Various Fish Species Landed at Selected Irish Ports, 2003. Abbotstown, Dublin:Marine Environment and Health Series.

USEPA (U.S. Environmental Protection Agency). 2004. Environmental Monitoring and Assessment Program (EMAP). Available: http://www.epa.gov/emap/ (EPA West) [accessed 26 April 2004].

USEPA (U.S. Environmental Protection Agency). 2005. Proceedings of the 2005 National Forum on Contaminants in Fish, Analysis of Chemical Contaminant Levels in Store-Bought Fish from Washington State. Available: http://water.epa.gov/scitech/swguidance/fishshellfish/techguidance/upload/2008_11_18_fish_forum_2005_proceedings2005.pdf [accessed 3 November 2011].

USEPA (U.S. Environmental Protection Agency). 2006a. Mid-Atlantic Integrated Assessment (MAIA). Available: http://www.epa.gov/emap/maia/html/data/estuary/9798/ [accessed 22 February 2006].

USEPA (U.S. Environmental Protection Agency). 2006b. Regional Environmental Monitoring and Assessment Program (REMAP). Available: http://www.epa.gov/emap/remap/html/data.html (Texas, 1993-1994) [accessed 21 June 2006].

USEPA (U.S. Environmental Protection Agency). 2007. Environmental Monitoring and Assessment Program (EMAP). Available: http://www.epa.gov/emap/ (Carolinian Province 1994-1997) [accessed 24 October 2007].

USEPA (U.S. Environmental Protection Agency). 2008. National Coastal Assessment. Available: http://www.epa.gov/emap/nca/html/data/index.html [accessed 3 October 2008].

USEPA (U.S. Environmental Protection Agency). 2011a. National Listing of Fish Advisories. Available: http://map1.epa.gov [accessed 14 September 2011].

USEPA (U.S. Environmental Protection Agency). 2011b. Environmental Monitoring and Assessment Program (EMAP). Available: http://www.epa.gov/emap/ (Virginian Province 1991-1993) [accessed 3 February 2011].

USEPA (U.S. Environmental Protection Agency). 2011c. Environmental Monitoring and Assessment Program (EMAP). Available: http://www.epa.gov/emap/ (Louisianian Province 1991-1994) [accessed 3 February 2011].

USEPA (U.S. Environmental Protection Agency) Region 9 and NOAA (National Oceanic and Atmospheric Administration. 2007. 2002-2004 Southern California Coastal Marine Fish Contaminants Survey. Available: http://earth1.epa.gov/region09/features/pvshelf/montrose_report.pdf [accessed 14 August 2007].

USFDA (U.S. Food and Drug Administration). 2011. Mercury Concentrations in Fish: FDA Monitoring Program. Available: http://www.fda.gov/Food/FoodSafety/Product-SpecificInformation/Seafood/FoodbornePathogensContaminants/Methylmercury/ucm191007.htm [accessed September 15, 2011].

USFDA (U.S. Food and Drug Administration). 2008. Total Diet Study 1991-2005. Available: http://www.fda.gov/downloads/Food/FoodSafety/FoodContaminantsAdulteration/TotalDietStudy/UCM243059.pdf [accessed 9 September 2008].

Usydus Z, Szlinder-Richert J, Polak-Juszczak L, Komar K, Adamczyk M, Malesa-Ciecwierz M, et al. 2009. Fish products available in Polish market - Assessment of the nutritive value and human exposure to dioxins and other contaminants. Chemosphere 74(11):1420-1428.

Vandenbroek WLF. 1981. Concentration and distribution of mercury in flesh of orange roughy (*Hoplostethus atlanticus*). N Z J Mar Freshw Res 15(3):255-260.

Vedrina-Dragojevic I, Dragojevic D, Bujan M. 2002. Total mercury content in fish und molluscs from Adriatic Sea. Dtsch Lebensm-Rundsch 98(1):10-13.

Viana F, Huertas R, Danulat E. 2005. Heavy metal levels in fish from coastal waters of Uruguay. Arch Environ Contam Toxicol 48(4):530-537.

Voegborlo RB, El-Methnani AM, Abedin MZ. 1999. Mercury, cadmium and lead content of canned tuna fish. Food Chem 67(4):341-345.

Voegborlo RB, Matsuyama A, Akagi H, Adimado AA, Ephraim JH. 2006. Total mercury and methylmercury accumulation in the muscle tissue of frigate (*Auxis thazard thazard*) and yellow fin (*Thunnus albacares*) tuna from the Gulf of Guinea, Ghana. Bull Environ Contam Toxicol 76(5):840-847.

Wang YW, Liang LN, Shi JB, Jiang GB. 2005. Chemometrics methods for the investigation of methylmercury and total mercury contamination in mollusks samples collected from coastal sites along the Chinese Bohai Sea. Environ Pollut 135(3):457-467.

Watling RJ, McClurg TP, Stanton RC. 1981. Relation between mercury concentration and size in the mako shark. Bull Environ Contam Toxicol 26(3):352-358.

Whyte ALH, Hook GR, Greening GE, Gibbs-Smith E, Gardner JPA. 2009. Human dietary exposure to heavy metals via the consumption of greenshell mussels (*Perna canaliculus* Gmelin 1791) from the Bay of Islands, northern New Zealand. Sci Total Environ 407(14):4348-4355.

Wren CD, Scheider WA, Wales DL, Muncaster BW, Gray IM. 1991. Relation between Mercury Concentrations in Walleye (*Stizostedion vitreum vitreum*) and Northern Pike (*Esox lucius*) in Ontario Lakes and Influence of Environmental-Factors. Can J Fish Aquat Sci 48(1):132-139.

Yamashita Y, Omura Y, Okazaki E. 2005. Total mercury and methylmercury levels in commercially important fishes in Japan. Fisheries Science 71(5):1029-1035.

Yamashita Y, Omura Y, Okazaki E. 2006. Distinct regional profiles of trace element content in muscle of Japanese eel *Anguilla japonica* from Japan, Taiwan, and China. Fisheries Science 72(5):1109-1113.

Zauke GP, Savinov VM, Ritterhoff J, Savinova T. 1999. Heavy metals in fish from the Barents Sea in (summer 1994). Sci Total Environ 227(2-3):161-173.

Zhang XM, Naidu AS, Kelley JJ, Jewett SC, Dasher D, Duffy LK. 2001. Baseline concentrations of total mercury and methylmercury in salmon returning via the Bering Sea (1999-2000). Mar Pollut Bull 42(10):993-997.
